# Assessment of Functional Mobility After COVID-19 in Adults Aged 50 Years or Older in the Canadian Longitudinal Study on Aging

**DOI:** 10.1001/jamanetworkopen.2021.46168

**Published:** 2022-01-12

**Authors:** Marla K. Beauchamp, Divya Joshi, Jacqueline McMillan, Urun Erbas Oz, Lauren E. Griffith, Nicole E. Basta, Susan Kirkland, Christina Wolfson, Parminder Raina

**Affiliations:** 1 School of Rehabilitation Science, Faculty of Health Sciences, McMaster University, Hamilton, Canada; 2 Labarge Centre for Mobility in Aging, McMaster University, Hamilton, Canada; 3 McMaster Institute for Research on Aging, McMaster University, Hamilton, Canada; 4 Department of Health Research Methods, Evidence, and Impact, Faculty of Health Sciences, McMaster University, Hamilton, Canada; 5 Department of Medicine, Section of Geriatric Medicine, University of Calgary, Calgary, Canada; 6 O’Brien Institute for Public Health, University of Calgary, Calgary, Canada; 7 Department of Epidemiology, Biostatistics and Occupational Health, McGill University, Montreal, Canada; 8 Department of Community Health & Epidemiology, Dalhousie University, Halifax, Canada; 9 Division of Geriatric Medicine, Department of Medicine, Dalhousie University, Halifax, Canada; 10 Department of Medicine, McGill University, Montreal, Canada; 11 Research Institute of the McGill University Health Centre, McGill University, Montreal, Canada; 12 Department of Psychiatry and Behavioral Neurosciences, McMaster University, Hamilton, Canada

## Abstract

**Importance:**

The association of COVID-19 not requiring hospitalization with functional mobility in community-dwelling adults above and beyond the impact of the pandemic control measures implemented in 2020 remains to be elucidated.

**Objective:**

To evaluate the association between a COVID-19 diagnosis and change in mobility and physical function of adults in Canada aged 50 years or older during the initial pandemic lockdown.

**Design, Setting, and Participants:**

This population-based cohort study used data from the Canadian Longitudinal Study on Aging (CLSA) COVID-19 study. This study was launched on April 15, 2020, and the exit questionnaires were completed between September and December 2020. Prepandemic data from the first CLSA follow-up (2015-2018) were also used. Respondents included middle-aged and older community-dwelling participants residing in Canadian provinces. Data were analyzed from February to May 2021.

**Exposures:**

The assessment for self-reported COVID-19 status was adapted from the Public Health Agency of Canada and the Centers for Disease Control and Prevention case definition available at the time of data collection; cases were classified as confirmed or probable, suspected, or non–COVID-19.

**Main Outcomes and Measures:**

Changes in mobility since the start of the COVID-19 pandemic were assessed using global rating of change in mobility scales at the COVID-19 exit questionnaire. Participant-reported new onset of difficulty in 3 physical function tasks was also examined.

**Results:**

Among 51 338 participants at baseline, 21 491 participants (41.9%) were 65 years or older and 26 155 participants (51.0%) were women and 25 183 (49.1%) were men. Of 2748 individuals with confirmed or probable or suspected COVID-19, 113 (94.2%) were not hospitalized. Individuals with confirmed or probable COVID-19 had higher odds of worsening mobility in terms of ability to engage in household activity (odds ratio [OR], 1.89; 95% CI, 1.11-3.22), physical activity (OR, 1.91; 95% CI, 1.32-2.76), and standing up after sitting in a chair (OR, 2.33; 95% CI, 1.06-5.11) compared with adults without COVID-19 during the same pandemic time period. Similar results were found for suspected COVID-19 status (eg, household activity: OR, 2.09; 95% CI, 1.82-2.41).

**Conclusions and Relevance:**

This cohort study among older adults in Canada found that receiving a COVID-19 diagnosis was significantly associated with worse mobility and functioning outcomes even in the absence of hospitalization. These findings suggest that interventions may be needed for individuals with mild to moderate COVID-19 who do not require hospitalization.

## Key Points

**Question:**

What is the association of a COVID-19 diagnosis and mobility and physical function among community-living middle-aged and older Canadians during the initial pandemic lockdown in 2020?

**Findings:**

This cohort study of 24 114 participants found that community-living middle-aged and older adults with confirmed, probable, or suspected COVID-19 had nearly 2-fold higher odds of worsening mobility and physical function compared with adults without COVID-19, although most participants with COVID-19 had mild to moderate disease and were not hospitalized.

**Meaning:**

These findings suggest that individuals with mild and moderate COVID-19 who were predominantly not hospitalized experienced deficits in functional mobility compared with those without COVID-19.

## Teaser

This cohort study evaluates the association of mild to moderate COVID-19 with change in mobility and physical function in a population-based sample of adults aged 50 years or older in Canada.

## Introduction

The World Health Organization (WHO) declared COVID-19 a pandemic on March 11, 2020, and as of December 2021, there have been more than 262 million confirmed cases and more than 5.2 million deaths globally.^1^ In response to COVID-19, local and national governments introduced multiple public health measures, including limitations and restrictions on sizes of gatherings, self-isolation and quarantine of known cases and close contacts, extended lockdowns, closure of nonessential services, and travel restrictions to prevent the spread of infection and mitigate its population health effects.^2^ Nevertheless, the ongoing pandemic and the associated public health measures have significant consequences for all populations and especially for older adults.^3^ Studies of clinical sequelae of COVID-19 in hospitalized cohorts have found an increased risk of neurological and psychiatric diagnoses and persistent physical and mental health symptoms associated with COVID-19; however, mobility and functioning outcomes are not well characterized. Even less is known about mobility and function in nonhospitalized samples of community-dwelling older adults.^4,5^

Mobility is well recognized as an essential component of well-being and an important determinant of healthy aging. A developing body of literature has shown that patients with severe COVID-19 who survived hospitalization report ongoing symptoms and physical limitations up to several months after discharge.^6,7,8,9^ There is also some emerging research to suggest that even mild to moderate COVID-19 can have negative ongoing functional associations for nonhospitalized patients.^10,11^ However, a major criticism of this early work is the use of convenience sampling (eg, recruitment via social media) and lack of control or comparison groups. In addition, the associations of preexisting sociodemographic and health characteristics in community-dwelling individuals with changes in mobility and functioning from COVID-19 remain to be elucidated. We hypothesized that individuals with confirmed or probable and suspected COVID-19 will be more likely to report worsening of mobility and physical function compared with those without COVID-19. The purpose of this study was to examine the association between a COVID-19 diagnosis and change in mobility and physical function among middle-aged and older adults who were enrolled in the Canadian Longitudinal Study on Aging (CLSA) COVID-19 study.

## Methods

This cohort study was approved by the Hamilton Integrated Research Ethics Board and by research ethics boards of all the participating institutions across Canada. Informed consent was obtained from the participants. This study follows the Strengthening the Reporting of Observational Studies in Epidemiology (STROBE) reporting guideline for cohort studies.

### Study Design and Participants

The CLSA consists of a national sample of 51 338 adults in Canada aged 45 to 85 years at the time of recruitment (baseline 2011-2015). Participants were recruited from across the 10 provinces and are followed-up every 3 years. Individuals residing in Canada’s 3 territories, on First Nation reserves, or in long-term care facilities, as well as members of the armed forces, those who were unable to communicate in English or French, and those with severe cognitive deficits were not eligible to participate in the study. A core set of information was collected from all participants by questionnaire. The first follow-up assessments were completed among 48 893 participants from 2015 to 2018. Further details on the CLSA study design and methods have been described elsewhere.^12,13^

In response to the onset of the COVID-19 pandemic, the CLSA launched the COVID-19 Questionnaire Study on April 15, 2020, to investigate the epidemiologic characteristics of COVID-19 among middle-aged and older adults. Of 51 338 CLSA participants, 42 700 were invited to participate. Overall, 42 511 participants were eligible to participate in the CLSA COVID-19 Study, of whom, 28 457 agreed (66.9%), and 24 114 (56.7%) completed the exit survey (September-December 2020) (eFigure 1 in Supplement 1). The CLSA COVID-19 Study collected longitudinal data over a 9-month period, with participants completing a 30-minute baseline questionnaire, four 10-minute weekly (34 498 participants who completed web-based questionnaire) or 2 biweekly (8202 participants who completed telephone-based interviews) questionnaires, 3 monthly questionnaires, and a 30-minute exit questionnaire. This study is based on complete data on outcomes and covariates from the CLSA first follow-up and cross-sectional data from the CLSA COVID-19 exit survey, as data on change in functional ability were collected at COVID-19 exit survey, and data on mobility were collected at the CLSA first follow-up and COVID-19 exit survey only. Characteristics of participants who did not participate in the COVID-19 Study vs those who participated are presented in eTable 1 in Supplement 1.

### COVID-19 Diagnosis

Assessment for self-reported COVID-19 diagnosis was based on data collected from all COVID-19 study questionnaires administered from baseline to exit, and was adapted from the Public Health Agency of Canada and the Centers for Disease Control and Prevention case definitions available at the time of data collection.^14,15^ The cases were classified as confirmed, probable, and suspected. Confirmed cases included participants who reported having been tested for COVID-19 (nucleic acid amplification test) and the results were positive. Probable cases included (1) participants who had exposure to a confirmed COVID-19 case and a body temperature greater than 38 °C or reported cough, who were tested for COVID-19, but the results were inconclusive; or (2) participants who reported having close contact with a confirmed COVID-19 case with either body temperature greater than 38 °C or reported cough; or (3) participants who were told by a health care practitioner that they had COVID-19 but they did not have a confirmatory test. We included this definition because early in the pandemic, testing was restricted to returning travelers, close contacts of known cases, and hospitalized patients. Suspected cases of COVID-19 included participants who reported exposure to a probable COVID-19 case and had 2 or more symptoms, including fever, cough, runny nose, sore or scratchy throat, headache, chills or shivering, muscle and/or joint aches and pains, loss of smell, or difficulty breathing. In the analysis, COVID-19 status was grouped as confirmed or probable, suspected, and no COVID-19. The exact timing of COVID-19 diagnosis in relation to mobility decline was not assessed in this study.

### Mobility and Physical Function

Changes in mobility since the start of the COVID-19 pandemic were assessed using global rating of change in mobility scales in the COVID-19 exit questionnaire. Consistent with the International Classification of Functioning, Disability and Health definition, participants were asked to report changes in their mobility in 3 domains: ability to move around the home, engage in housework, and engage in physical activity on a 5-point Likert scale ranging from 1 indicating much worse to 5, much better. In this analysis, each outcome was dichotomized into either worse or not worse. Self-reported physical function for 3 specific functional tasks was assessed at CLSA first follow-up and the COVID-19 exit survey. Participants were asked to report whether or not they experienced difficulty in standing up after sitting in a chair, walking up and down a flight of stairs without assistance, and walking 2 to 3 neighborhood blocks. Participants who experienced difficulty were further probed about the degree of difficulty, with response options being a little difficult, somewhat difficult, and very difficult (eTable 2 in Supplement 1). Responses for each item from the CLSA first follow-up and the COVID-19 exit questionnaire were compared to create a change variable with categories of worse and not worse.

### Assessment of Covariates

Covariates included age at COVID-19 exit questionnaire and prepandemic variables from CLSA first follow-up, including sex, annual household income (categorized as <CAD$50 000, CAD$50 000 to <CAD$100 000, and ≥CAD$100 000), dwelling type (house including single detached, semidetached, duplex or townhouse; apartment or condominium; or other form of housing, including seniors’ housing, institution, mobile home, hotel, rooming or lodging house), living area (urban or rural), smoking (never, former, or current smoker), physical activity (adequate or low activity), nutritional intake (high risk or not at risk), and number of chronic conditions from 10 disease categories, including musculoskeletal, respiratory, cardiovascular, endocrine-metabolic, neurological, gastrointestinal, genitourinary, ophthalmologic, renal, and cancer, which were added and categorized as less than 3 or 3 or more conditions. Race and ethnicity was self-reported, and it was included as a covariate in the sensitivity analysis.

The Physical Activity Scale for Elderly was used to assess participants’ physical activity levels.^16^ Based on the World Health Organization’s age-specific recommendations, adequate physical activity was classified as engaging in at least 150 minutes of moderate-intensity or at least 75 minutes of vigorous-intensity physical activity per week.^17^ Nutritional behavior was assessed using the Seniors in the Community: Risk Evaluation for Eating and Nutrition tool.^18^ Using previously validated cutoff of a score less than 32 was identified as high risk.^18^

### Statistical Analysis

Descriptive statistics for all participants were presented as frequency and percentage. CLSA first follow-up data were used to impute a variable that was not assessed in the COVID-19 baseline and exit surveys, and COVID-19 baseline data were used to impute a variable that was not assessed in the COVID-19 exit survey. Multivariable logistic regression models were used to examine the association between COVID-19 status and change in mobility and physical function outcomes. All multivariable models were adjusted for the prepandemic covariates, since they are known to be associated with increased risk of COVID-19 and poor functional mobility outcomes. Unadjusted and adjusted odds ratios (aORs) and 95% CIs were reported. Statistical analyses were conducted using SAS software version 9.4 (SAS Institute). The proportion of worsening of the mobility and function outcomes among the COVID-19 exposure groups were similar under conditions of missing covariates and in the complete case scenario (eTable 3 in Supplement 1). Statistical significance was set at 2-tailed α = .05. Data were analyzed from February to May 2021.

## Results

Among 51 338 participants at CLSA baseline, 21 491 participants (41.9%) were aged 65 years or older, 26 155 (51.0%) were women, and 25 183 (49.1%) were men. According to self-report, 47 105 participants (92.7%) were of European ethnic background and 3718 participants (7.3%) were of non-European ethnic background, including Southeast Asian, South Asian, West Asian, Chinese, Japanese, Korean, Black, Latin American, Filipino, Indigenous populations (Table 1). Descriptive characteristics of study participants by COVID-19 status are presented in eTable 4 in Supplement 1. Of these, 29 559 participants responded to the baseline COVID-19 questionnaire. Based on data collected from all COVID-19 Study questionnaires, 159 participants (0.6%) were classified as having probable or confirmed COVID-19 and 2589 participants (9.4%) were classified as having suspected COVID-19. Of 24 114 participants who completed the COVID-19 exit questionnaire, 121 participants (0.6%) either had a positive COVID-19 test result (70 participants) or were diagnosed with COVID-19 by a health care practitioner but did not have a confirmatory test (51 participants). Of these 121 participants, 7 participants (5.8%) reported an inpatient hospital stay when asked to report on the type of care or treatment they had received after their COVID-19 diagnosis, and 113 nonhospitalized participants (94.2%) were considered to have had a mild to moderate form of the disease.

**Table 1.** Distribution of Sociodemographic and Health Factors in the CLSA Baseline and First Follow-up and at COVID-19 Baseline and Exit Time Points

Characteristic	CLSA, No. (%)			
	Baseline (2011-2015) (n = 51 338)	First follow-up (2015-2018) (n = 44 817)	COVID-19	
			Baseline (April 15-May 30, 2020) (n = 28 559	Exit (September 29-December 30, 2020) (n = 24 114)
Age group, y				
<65	29 847 (58.1)	21 349 (47.6)	10 465 (36.7)	8026 (33.3)
≥65	21 491 (41.9)	23 468 (52.4)	18 094 (63.4)	16 088 (66.7)
Sex				
Women	26 155 (51.0)	22 944 (51.2)	14 982 (52.5)	12 819 (53.2)
Men	25 183 (49.1)	21 873 (48.8)	13 577 (47.5)	11 295 (46.8)
Ethnicity				
European	47 105 (92.7)	41 273 (93.0)	26 487 (93.5)	22 439 (93.8)
Non-European^a^	3718 (7.3)	3132 (7.1)	1844 (6.5)	1485 (6.2)
Annual household income, CAD$^b^^,^^c^				
<$50 000	15 122 (31.5)	12 012 (28.9)	6735 (25.2)	5716 (25.3)
$50 000 to <$100 000	17 127 (35.7)	15 124 (36.4)	10 014 (37.4)	8571 (37.9)
≥$100 000	15 778 (32.9)	14 426 (34.7)	10 005 (37.4)	8347 (36.9)
Dwelling type^d^				
House	41 759 (81.4)	35 549 (79.3)	22 201 (77.9)	18 740 (77.8)
Apartment or condominium	8816 (17.2)	8006 (17.9)	5201 (18.2)	4434 (18.4)
Other	746 (1.5)	1259 (2.8)	1111 (3.9)	910 (3.8)
Living area^d^				
Rural	9634 (18.8)	6660 (14.9)	5114 (17.9)	4278 (17.8)
Urban	41 704 (81.2)	38 126 (85.1)	23 297 (81.6)	19 706 (82.2)
No. of chronic conditions^b^				
<3	33 707 (68.3)	22 776 (52.9)	15 042 (54.9)	12 633 (54.3)
≥3	15 618 (31.7)	20 308 (47.1)	12 369 (45.1)	10 532 (45.5)
Smoking^d^				
Current	4843 (9.5)	3285 (7.4)	1790 (6.5)	1448 (6.1)
Former	30 530 (59.8)	27 313 (61.3)	17 176 (61.9)	14 729 (62.2)
Never	15 684 (30.7)	13 961 (31.3)	8767 (31.6)	7523 (31.7)
Physical activity^b^				
Adequate activity	14 114 (29.5)	12 988 (29.0)	8966 (31.6)	7555 (31.5)
Low activity	33 713 (70.5)	31 807 (71.0)	19 392 (68.4)	16 414 (68.5)
Nutritional intake				
High risk	5649 (12.2)	6517 (15.2)	3733 (13.6)	3018 (13.0)
Not at risk	40 772 (87.8)	36 296 (84.8)	23 784 (86.4)	20 238 (87.0)

Abbreviation: CLSA, Canadian Longitudinal Study on Aging.

^a^ Non-European ethnic backgrounds included Southeast Asian, South Asian, West Asian, Chinese, Japanese, Korean, Black, Latin American, Filipino, Indigenous populations, and other ethnicities.

^b^ Data for annual household income, number of chronic conditions, and physical activity variables were not collected in COVID-19 baseline and exit surveys, so we used CLSA follow-up 1 values for the COVID-19 sample.

^c^ Income groups in US dollars are less than $39 097.32, $39 097.32 to less than $78 194.64, and $78 194.64 or more.

^d^ Data for dwelling type, living area, and smoking status were not collected in COVID-19 exit questionnaire, so we used COVID-19 baseline values for the COVID-19 exit sample.

During the COVID-19 pandemic, more than 1 in 4 participants (5976 participants [25.2%]) reported worsening ability to engage in physical activity (1349 participants [5.7%] reported much worse ability), 2111 participants (8.9%) reported worsening ability to move around in their home (338 participants [1.4%] reported much worse ability), and 2022 participants (8.6%) reported worsening ability to engage in housework (336 participants [1.6%] reported much worse ability). Compared with rates reported at the first CLSA follow-up, 3639 participants (15.2%) reported new difficulty in standing up after sitting in a chair, 2489 participants (10.4%) reported new difficulty walking up and down a flight of stairs without assistance, and 2656 participants (11.1%) reported new difficulty walking 2 to 3 neighborhood blocks.

### Association Between COVID-19 Status and Change in Mobility and Physical Function

Compared with individuals without COVID-19, individuals with probable or confirmed COVID-19 had nearly 2-fold higher odds of reporting worsening ability to engage in household activity (aOR, 1.89; 95% CI, 1.11-3.22) and participate in physical activity (aOR, 1.91; 95% CI, 1.32-2.76), after adjusting for covariates. Similarly, individuals with suspected COVID-19 were also 2-fold as likely to report worsening ability to move around in the home (aOR, 2.30; 95% CI, 2.01-2.63), engage in housework activity (aOR, 2.09; 95% CI, 1.82-2.41), and participate in physical activity (aOR, 1.78; 95% CI, 1.61-1.97) compared with individuals without COVID-19 (Table 2; eFigure 2 in Supplement 1).

**Table 2.** Multivariable Logistic Regression Models Examining the Association Between COVID-19 and Worsening Mobility Since the Start of the Pandemic

Model	OR (95% CI)^a^		
	Ability to move around in home (n = 20 386)^b^	Ability to engage in housework activity (n = 20 361)^c^	Ability to engage in physical activity (n = 20 426)^d^
**Unadjusted **			
COVID-19 Status			
No COVID-19	1 [Reference]	1 [Reference]	1 [Reference]
Probable or confirmed COVID-19	1.90 (1.18-3.06)	2.10 (1.32-3.35)	2.16 (1.55-3.02)
Suspected COVID-19	2.63 (2.33-2.96)	2.56 (2.26-2.89)	1.97 (1.80-2.16)
**Adjusted^e^**			
COVID-19 status			
No COVID-19	1 [Reference]	1 [Reference]	1 [Reference]
Probable or confirmed COVID-19	1.60 (0.92-2.79)	1.89 (1.11-3.22)	1.91 (1.32-2.76)
Suspected COVID-19	2.30 (2.01-2.63)	2.09 (1.82-2.41)	1.78 (1.61-1.97)
Age ≥65 y (vs age <65 y)	1.47 (1.29-1.67)	1.19 (1.04-1.35)	1.05 (0.97-1.13)
Female sex (vs male sex)	1.02 (0.92-1.13)	1.50 (1.34-1.67)	1.20 (1.12-1.28)
Annual household income, CAD$^f^			
<$50 000	1.19 (1.03-1.38)	1.50 (1.29-1.74)	0.90 (0.82-0.99)
$50 000 to <$100 000	1.14 (1.00-1.30)	1.22 (1.06-1.40)	0.99 (0.92-1.07)
≥$100 000	1 [Reference]	1 [Reference]	1 [Reference]
Dwelling type			
House	1 [Reference]	1 [Reference]	1 [Reference]
Apartment or condominium	1.20 (1.05-1.36)	1.27 (1.12-1.44)	1.24 (1.13-1.35)
Other	1.05 (0.76-1.45)	1.06 (0.77-1.47)	1.06 (0.84-1.34)
Urban residence (vs rural)	1.10 (0.95-1.28)	1.16 (0.99-1.36)	1.33 (1.20-1.47)
≥3 chronic conditions (vs <3 chronic conditions)	2.27 (2.03-2.55)	2.16 (1.92-2.43)	1.55 (1.45-1.67)
Smoking status			
Current	1.27 (1.02-1.58)	1.48 (1.20-1.83)	0.99 (0.86-1.15)
Former	1.12 (1.00-1.26)	1.16 (1.03-1.31)	1.05 (0.98-1.12)
Never	1 [Reference]	1 [Reference]	1 [Reference]
Low physical activity (vs adequate activity)	1.27 (1.13-1.44)	1.30 (1.15-1.48)	0.91 (0.85-0.98)
High nutritional risk (vs not at risk)	1.88 (1.66-2.14)	2.20 (1.94-2.50)	1.58 (1.44-1.73)

Abbreviations: CLSA, Canadian Longitudinal Study on Aging; OR, odds ratio.

^a^ Change in mobility since the start of the pandemic was assessed based on the data collected at the COVID-19 exit survey. Sample size is reported for the fully adjusted model.

^b^ COVID-19 status and worsening of the ability to move around in the home (adjusted model) included 1369 participants without COVID-19, 350 participants with suspected COVID-19, and 15 participants with confirmed or probable COVID-19.

^c^ COVID-19 status and worsening of the ability to engage in housework activity (adjusted model) included 1307 participants without COVID-19, 317 participants with suspected COVID-19, and 17 participants with confirmed or probable.

^d^ COVID-19 status and worsening of the ability to engage in physical activity (adjusted model) included 4283 participants without COVID-19, 724 participants with suspected COVID-19, and 48 participants with confirmed or probable COVID-19.

^e^ The associations were adjusted for age, sex, annual household income, dwelling type, living area, number of chronic conditions, smoking status, physical activity, and nutritional risk.

^f^ Income groups in US dollars are less than $39 097.32, $39 097.32 to less than $78 194.64, and $78 194.64 or more.

After adjusting for covariates, individuals with confirmed or probable COVID-19 were more likely to experience worsening difficulty standing up after sitting in a chair (aOR, 2.33; 95% CI, 1.06-5.11), as were those with suspected COVID-19 (aOR, 1.70; 95% CI, 1.37-2.10). Furthermore, individuals with suspected COVID-19 were also more likely to experience worsening difficulty with walking up and down a flight of stairs (aOR, 1.95; 95% CI, 1.55-2.44) and walking 2 to 3 neighborhood blocks (aOR, 1.80; 95% CI, 1.45-2.22) compared with those without COVID-19 (Table 3; eFigure 2 in Supplement 1). We also tested models after additionally adjusting for ethnicity, number of people living in the same household, social participation, and alcohol consumption. These variables did not have any significant associations and did not confound the association between COVID-19 and mobility and functioning; thus, they were subsequently excluded (eTable 5 and eTable 6 in Supplement 1). We also explored interactions of COVID-19 status with age, sex, household income, and number of chronic conditions (eTables 7-12 in Supplement 1). Overall, participants with COVID-19 characterized by older age, lower total income, living in an apartment or condominium rather than a house, having 3 or more chronic conditions, inadequate physical activity, and poor nutritional intake also reported worsening for many of the mobility and functioning outcomes (Tables 2 and 3)

**Table 3.** Multivariable Logistic Regression Models Examining the Association Between COVID-19 and Worsening Physical Function

Model	OR (95% CI)^a^		
	Difficulty standing up after sitting in a chair (n = 7731)^b^	Difficulty walking alone up and down a flight of stairs (n = 7723)^c^	Difficulty walking 2-3 neighborhood blocks (n = 7700)^d^
**Unadjusted model**			
COVID-19 status			
No COVID-19	1 [Reference]	1 [Reference]	1 [Reference]
Probable or confirmed COVID-19	1.98 (0.96-4.08)	1.49 (0.64-3.52)	0.98 (0.39-2.47)
Suspected COVID-19	1.82 (1.49-2.22)	2.24 (1.83-2.73)	2.08 (1.72-2.52)
**Adjusted model^e^**			
COVID-19 Status			
No COVID-19	1 [Reference]	1 [Reference]	1 [Reference]
Probable or confirmed COVID-19	2.33 (1.06-5.11)	1.55 (0.58-4.13)	1.00 (0.34-2.93)
Suspected COVID-19	1.70 (1.37-2.10)	1.95 (1.55-2.44)	1.80 (1.45-2.22)
Age ≥65 y (vs <65 y)	1.26 (1.05-1.52)	1.96 (1.55-2.48)	1.56 (1.27-1.92)
Female sex (vs male sex)	0.93 (0.79-1.08)	1.01 (0.85-1.20)	0.98 (0.83-1.15)
Annual household income, CAD$^f^			
<$50 000	1.48 (1.19-1.83)	1.84 (1.44-2.35)	2.35 (1.86-2.98)
$50 000 to <$100 000	1.22 (1.00-1.49)	1.37 (1.08-1.74)	1.77 (1.41-2.22)
≥$100 000	1 [Reference]	1 [Reference]	1 [Reference]
Dwelling type			
House	1 [Reference]	1 [Reference]	1 [Reference]
Apartment or condominium	0.96 (0.78-1.19)	1.33 (1.07-1.64)	1.07 (0.87-1.32)
Other	0.87 (0.57-1.34)	1.61 (1.10-2.36)	1.28 (0.88-1.85)
Urban residence (vs rural)	0.96 (0.80-1.14)	1.03 (0.85-1.26)	0.96 (0.80-1.15)
≥3 chronic conditions (vs <3 chronic conditions)	1.75 (1.49-2.06)	2.38 (1.97-2.87)	3.01 (2.52-3.59)
Smoking status			
Current	1.08 (0.78-1.50)	1.11 (0.78-1.58)	1.20 (0.88-1.66)
Former	1.16 (0.98-1.38)	1.20 (0.99-1.45)	1.11 (0.93-1.32)
Never	1 [Reference]	1 [Reference]	1 [Reference]
Low physical activity (vs adequate activity)	1.29 (1.07-1.55)	1.56 (1.25-1.94)	1.55 (1.26-1.89)
High nutritional risk (vs not at risk)	1.52 (1.24-1.86)	1.60 (1.29-1.97)	1.83 (1.51-2.22)

Abbreviations: CLSA, Canadian Longitudinal Study on Aging; OR, odds ratio.

^a^ Change in physical function was assessed based on the data collected at CLSA first follow-up and the COVID-19 exit survey. The sample size reflects the number of participants who provided data at both these time points and is reported for the fully adjusted model.

^b^ COVID-19 status and worsening difficulty standing up after sitting in a chair (adjusted model) included 657 participants without COVID-19, 123 participants with suspected COVID-19, and 8 with confirmed or probable COVID-19.

^c^ COVID-19 status and worsening difficulty walking alone up and down a flight of stairs (adjusted model) included 437 participants without COVID-19, 119 participants with suspected COVID-19, and 5 participants with confirmed or probable COVID-19.

^d^ COVID-19 status and worsening difficulty walking 2 to 3 neighborhood blocks (adjusted model) included 656 participants without COVID-19, 137 participants with suspected COVID-19, and 4 participants with confirmed or probable COVID-19.

^e^ The associations were adjusted for age, sex, annual household income, dwelling type, living area, number of chronic conditions, smoking status, physical activity, and nutritional risk.

^f^ Income groups in US dollars are less than $39 097.32, $39 097.32 to less than $78 194.64, and $78 194.64 or more.

## Discussion

This cohort study is the first study, to our knowledge, to evaluate the associations of confirmed, probable, or suspected COVID-19 with the mobility and functioning of a community-based sample of middle-aged and older adults. Our results showed that community-dwelling individuals who reported confirmed, probable, or suspected COVID-19 had higher odds of worsening mobility since the start of the pandemic and worsening physical function since the CLSA first follow-up compared with those without COVID-19. Importantly, in a population-based study with a built-in comparison group, these findings highlight the burden of mild to moderate COVID-19 not requiring hospitalization on physical health in community-living people.

Although there is a growing body of research showing that hospitalized patients with COVID-19 experience problems with physical functioning up to 6 months after discharge, there is still a dearth of literature on nonhospitalized patients with less severe illness.^5,6,19,20^ Anecdotal reports, patient accounts on social media, and some preliminary research with convenience samples, have suggested that many patients who experience even mild COVID-19 have persistent and troublesome symptoms, including impaired physical function after their initial illness.^10,11,21^ There is an ongoing effort by both health professionals and patients alike to recognize long COVID as a long-term condition and to increase access to treatments and rehabilitative care.^22,23,24^ Our findings confirm that individuals with COVID-19 who did not require hospitalization were more likely than those without COVID-19 to experience worsening of overall mobility since the start of the pandemic and a deterioration in physical function at the COVID-19 exit survey compared with the CLSA first follow-up. Research suggests certain coronaviruses can cause inflammatory damage to the central nervous system tissue.^25^ Studies conducted in individuals with severe acute respiratory syndrome and Middle East respiratory syndrome reported prolonged fatigue, sleep disturbances, and changes in cognition after recovery from infection.^26,27,28^ Similar symptoms have been reported by individuals after COVID-19.^28,29,30^ Some evidence suggests that the SARS-CoV-2 can cause neuroinflammation and inflammation in other parts of the body, resulting in neuronal degeneration and release of proinflammatory cytokines, which may explain the subsequent chronic fatigue and functional mobility impacts experienced by many individuals after COVID-19.^28,29,30^ It is also possible that public health recommendations for quarantine and self-isolation for individuals who have test results positive for COVID-19 restricted physical activity and may have exacerbated the mobility and physical function decline.^31,32^

Further, our results showed that sociodemographic risk factors and having 3 or more chronic conditions were associated with a decline in mobility and/or functioning. These risk factors have been associated with severe COVID-19 and its complications,^33,34^ and our findings indicate that these risk factors are also negatively associated with physical health outcomes in community-dwelling individuals, some of whom may have mild to moderate COVID-19. Therefore, older adults in these subgroups who may become ill with COVID-19 should be prioritized when planning interventions. Taken together with previous work, our results suggest a need for approaches to effectively restore functional mobility to predisease levels after COVID-19. It is recommended that approaches that promote gradual activity and enhance social, cultural, and financial support may help with managing post–COVID-19 conditions.^35^

The strengths of this study include the timing of data collection in the midst of the COVID-19 pandemic. Additionally, our use of a large sample of community-living adults to examine changes in function and mobility relative to a COVID-19 diagnosis make our findings more nationally generalizable.

### Limitations

Our study has several limitations that should be considered when interpreting the findings. COVID-19 status was classified based on self-reports, and not all cases were confirmed with testing. The number of participants with confirmed or probable COVID-19 was small, which may have resulted in lower statistical power to examine associations for this group. Furthermore, the exact timing of COVID-19 diagnosis in relation to mobility decline or duration of functional mobility deficits was not assessed in our study. Functional mobility was not assessed using performance-based tests and therefore the mobility outcomes may be prone to recall bias. Additionally, individuals at risk of experiencing worsening of mobility may be more likely to withdraw from the study, which may underestimate the associations. A greater proportion of participants who did not participate in the COVID-19 study were older and had lower income, which may impact the validity of our findings. Additionally, this study did not include individuals residing in long-term care institutions, who were more often hospitalized with COVID-19 and whose change in function or mobility associated with a COVID-19 diagnosis may be different than that experienced by community-dwelling older adults. In Canada, health measures taken by the governments and hospitals included creating additional critical care beds; in regions where hospitals were overwhelmed with COVID-19 admissions, some critical care patients were transferred to other hospital locations with increased capacity.^36^ Thus, it is important to note that the low number of hospitalizations in our study is not a reflection that participants were turned away from hospitalization and sent home, but rather a reflection of the CLSA sample of community-dwelling middle aged and older adults.

## Conclusions

In this population-based cohort study of community-dwelling middle-aged and older adults, we found that mild to moderate COVID-19 was associated with worsening mobility and difficulties in physical functioning. In view of the large number of adults diagnosed with COVID-19 worldwide, there is a need to further understand the longer-term impacts of the illness and to consider the development and implementation of effective intervention and management approaches to address any persistent deficits in mobility and functioning among those living in the community.

## Notes

Supplement 1. **eFigure 1.** Flowchart of Study Participants in the CLSA COVID-19 Study **eTable 1.** Characteristics of Participants Who Did Not Participate in the COVID-19 Study and Those Who Did **eTable 2.** Items Used to Assess Change in Mobility and Physical Function **eTable 3.** Distribution of the Mobility and Function Outcomes by COVID-19 Exposure Status in the Presence of Missing Covariates and in Complete Case Analyses **eTable 4.** Descriptive Characteristics of Study Participants by COVID-19 Status in the CLSA COVID-19 Study **eFigure 2.** Multivariable Analysis of the Association of COVID-19 With Worsening Mobility and Physical Function **eTable 5.** Multivariable Analysis of the Association of COVID-19 With Worsening of Mobility After Adjusting for Additional Covariates **eTable 6.** Multivariable Analysis of the Association of COVID-19 With Worsening Physical Function After Adjusting for Additional Covariates **eTable 7.** Multivariable Logistic Regression Models of the Association Between COVID-19 and Worsening Ability to Move Around in Home Since the Start of the Pandemic **eTable 8.** Multivariable Logistic Regression Models of the Association Between COVID-19 and Worsening Ability to Engage in Household Activity Since the Start of the Pandemic **eTable 9.** Multivariable Logistic Regression Models of the Association Between COVID-19 and Worsening Ability to Engage in Physical Activity Since the Start of the Pandemic **eTable 10.** Multivariable Logistic Regression Models of the Association Between COVID-19 and Worsening Difficulty Standing Up After Sitting in a Chair **eTable 11.** Multivariable Logistic Regression Models of the Association Between COVID-19 and Worsening Difficulty Walking Alone Up and Down a Flight of Stairs **eTable 12.** Multivariable Logistic Regression Models of the Association Between COVID-19 and Worsening Difficulty Walking 2 to 3 Neighborhood Blocks

Supplement 2.
The Canadian Longitudinal Study on Aging Team Members
