# Errors in Abstract, Main Text, and Supplement 1

**DOI:** 10.1001/jamanetworkopen.2022.0927

**Published:** 2022-02-14

In the Original Investigation titled “Assessment of Functional Mobility After COVID-19 in Adults Aged 50 Years or Older in the Canadian Longitudinal Study on Aging,”^1^ published January 12, 2022, there were errors in the presentation of data regarding patients hospitalized with confirmed or probable COVID-19 in the Abstract and in the cohort size in the main text and eTable 4 in Supplement 1. This article has been corrected.^1^
