# Supplementary material for: Assessment of Functional Mobility After COVID-19 in Adults Aged 50 Years or Older in the Canadian Longitudinal Study on Aging
Source: JAMA Netw Open. 2022 Jan 12;5(1):e2146168. doi: 10.1001/jamanetworkopen.2021.46168 (PMC8756318; doi:10.1001/jamanetworkopen.2021.46168)
Supplement: Supplement 1. — eFigure 1. Flowchart of Study Participants in the CLSA COVID-19 Study eTable 1. Characteristics of Participants Who Did Not Participate in the COVID-19 Study and Those Who Did eTable 2. Items Used to Assess Change in Mobility and Physical Function eTable 3. Distribution of the Mobility and Function Outcomes by COVID-19 Exposure Status in the Presence of Missing Covariates and in Complete Case Analyses eTable 4. Descriptive Characteristics of Study Participants by COVID-19 Status in the CLSA COVID-19 Study eFigure 2. Multivariable Analysis of the Association of COVID-19 With Worsening Mobility and Physical Function eTable 5. Multivariable Analysis of the Association of COVID-19 With Worsening of Mobility After Adjusting for Additional Covariates eTable 6. Multivariable Analysis of the Association of COVID-19 With Worsening Physical Function After Adjusting for Additional Covariates eTable 7. Multivariable Logistic Regression Models of the Association Between COVID-19 and Worsening Ability to Move Around in Home Since the Start of the Pandemic eTable 8. Multivariable Logistic Regression Models of the Association Between COVID-19 and Worsening Ability to Engage in Household Activity Since the Start of the Pandemic eTable 9. Multivariable Logistic Regression Models of the Association Between COVID-19 and Worsening Ability to Engage in Physical Activity Since the Start of the Pandemic eTable 10. Multivariable Logistic Regression Models of the Association Between COVID-19 and Worsening Difficulty Standing Up After Sitting in a Chair eTable 11. Multivariable Logistic Regression Models of the Association Between COVID-19 and Worsening Difficulty Walking Alone Up and Down a Flight of Stairs eTable 12. Multivariable Logistic Regression Models of the Association Between COVID-19 and Worsening Difficulty Walking 2 to 3 Neighborhood Blocks [file jamanetwopen-e2146168-s001.pdf]

Supplemental Online Content

Beauchamp MK, Joshi D, McMillan J, et al; Canadian Longitudinal Study on Aging (CLSA) Team. Assessment of functional mobility after COVID-19 in adults aged 50 years or older in the Canadian Longitudinal Study on Aging. *JAMA Netw Open*. 2022;5(1):e2146168. doi:10.1001/jamanetworkopen.2021.46168

eFigure 1. Flowchart of Study Participants in the CLSA COVID-19 Study
eTable 1. Characteristics of Participants Who Did Not Participate in the COVID-19 Study and Those Who Did
eTable 2. Items Used to Assess Change in Mobility and Physical Function
eTable 3. Distribution of the Mobility and Function Outcomes by COVID-19 Exposure Status in the Presence of Missing Covariates and in Complete Case Analyses
eTable 4. Descriptive Characteristics of Study Participants by COVID-19 Status in the CLSA COVID-19 Study
eFigure 2. Multivariable Analysis of the Association of COVID-19 With Worsening Mobility and Physical Function
eTable 5. Multivariable Analysis of the Association of COVID-19 With Worsening of Mobility After Adjusting for Additional Covariates
eTable 6. Multivariable Analysis of the Association of COVID-19 With Worsening Physical Function After Adjusting for Additional Covariates
eTable 7. Multivariable Logistic Regression Models of the Association Between COVID-19 and Worsening Ability to Move Around in Home Since the Start of the Pandemic
eTable 8. Multivariable Logistic Regression Models of the Association Between COVID-19 and Worsening Ability to Engage in Household Activity Since the Start of the Pandemic
eTable 9. Multivariable Logistic Regression Models of the Association Between COVID-19 and Worsening Ability to Engage in Physical Activity Since the Start of the Pandemic
eTable 10. Multivariable Logistic Regression Models of the Association Between COVID-19 and Worsening Difficulty Standing Up After Sitting in a Chair
eTable 11. Multivariable Logistic Regression Models of the Association Between COVID-19 and Worsening Difficulty Walking Alone Up and Down a Flight of Stairs
eTable 12. Multivariable Logistic Regression Models of the Association Between COVID-19 and Worsening Difficulty Walking 2 to 3 Neighborhood Blocks

This supplemental material has been provided by the authors to give readers additional information about their work.

© 2022 Beauchamp MK et al. *JAMA Network Open.*

eFigure 1. Flowchart of Study Participants in the CLSA COVID-19 Study

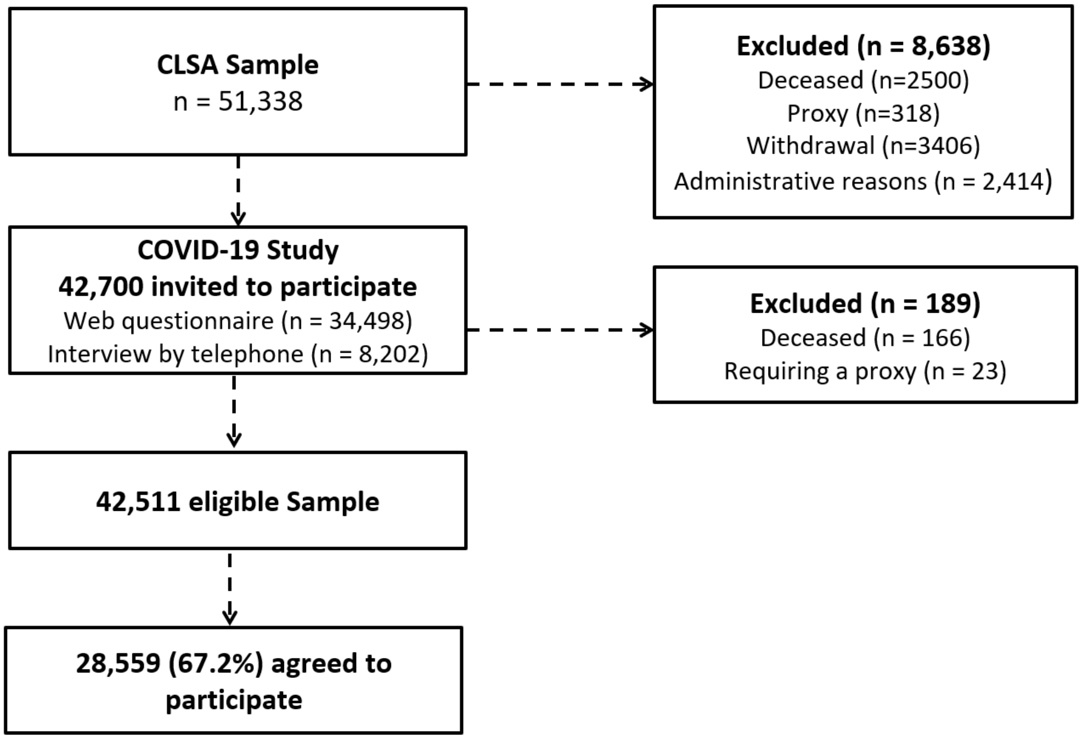

© 2022 Beauchamp MK et al. *JAMA Network Open.*

eTable 1. Characteristics of Participants Who Did Not Participate in the COVID-19 Study and Those Who Did

	Did not participate in COVID-19 Study				Participated in COVID-19 Study			
	CLSA Baseline (22,779)		CLSA First Follow-up (16,452)		CLSA Baseline (28,365)		CLSA First Follow-up (28,365)	
	n	%	n	%	n	%	n	%
**Age Group**								
<55	5887	25.84	2533	15.40	7460	26.30	4065	14.33
55-64	6319	27.74	4890	29.72	10044	35.41	9861	34.76
65-74	5010	21.99	4383	26.64	6953	24.51	8919	31.44
75+	5563	24.42	4646	28.24	3908	13.78	5520	19.46
**Sex**								
Female	11173	49.05	8066	49.03	14878	52.45	14878	52.45
Male	11606	50.95	8386	50.97	13487	47.55	13487	47.55
**Ethnicity**								
White	20618	91.67	14960	91.98	26313	93.51	26313	93.51
Non-White	1874	8.33	1305	8.02	1827	6.49	1827	6.49
**Annual Household Income**								
Less than $20,000	1832	8.70	1055	7.12	1064	3.97	1028	3.84
$20,000 or more, but less than $50,000	6465	30.70	4222	28.51	5706	21.31	5707	21.33
$50,000 or more, but less than $100,000	7162	34.01	5110	34.51	9908	37.00	10014	37.43
$100,000 or more, but less than $150,000	3231	15.34	2416	16.32	5465	20.41	5394	20.16
$150,000 or more	2369	11.25	2005	13.54	4638	17.32	4611	17.23
**Number of People Living in the Same Household**								
Living alone	5879	25.83	4211	26.87	5833	20.57	6493	22.95
Not living alone	16880	74.17	11458	73.13	22525	79.43	21803	77.05
**Dwelling Type**								
House (single detached, semi-detached, duplex or townhouse)	17855	78.41	12634	76.8	23740	83.72	22915	80.79
Apartment or condominium	4433	19.47	3140	19.09	4355	15.36	4866	17.16
Other	482	2.12	677	4.12	262	0.92	582	2.05
**Living Area**								
Rural	4788	21.02	2681	16.31	4806	16.94	3979	14.04
Urban	17991	78.98	13756	83.69	23559	83.06	24370	85.96

© 2022 Beauchamp MK et al. *JAMA Network Open.*

eTable 2. Items Used to Assess Change in Mobility and Physical Function

1. Your ability to move around in your home (such as walking, climbing stairs) has become …	Much worse<br>A little bit worse<br>Stayed about the same<br>A little bit better<br>Much better<br>Don't know/No answer<br>Prefer not to answer	
2. Your ability to engage in housework activity (such as dusting, washing dishes, and vacuuming) has become …	Much worse<br>A little bit worse<br>Stayed about the same<br>A little bit better<br>Much better<br>Don't know/No answer<br>Prefer not to answer	
3. Your ability to engage in physical activity (walking, exercise, working out) has become…	Much worse<br>A little bit worse<br>Stayed about the same<br>A little bit better<br>Much better<br>Don't know/No answer<br>Prefer not to answer	
4. Do you have any difficulty standing up after sitting in a chair?	Yes<br>No<br>Unable to do<br>Don't know/No answer<br>Prefer not to answer	If yes,<br>A little difficult<br>Somewhat difficult<br>Very difficult<br>Don't know/No answer<br>Prefer not to answer
5. Do you have any difficulty walking alone up and down a flight of stairs?	Yes<br>No<br>Unable to do<br>Don't know/No answer<br>Prefer not to answer	If yes,<br>A little difficult<br>Somewhat difficult<br>Very difficult<br>Don't know/No answer<br>Prefer not to answer
6. Do you have any difficulty walking 2 to 3 neighbourhood blocks?	Yes<br>No<br>Unable to do<br>Don't know/No answer<br>Prefer not to answer	If yes,<br>A little difficult<br>Somewhat difficult<br>Very difficult<br>Don't know/No answer<br>Prefer not to answer

© 2022 Beauchamp MK et al. *JAMA Network Open.*

eTable 3. Distribution of the Mobility and Function Outcomes by COVID-19 Exposure Status in the Presence of Missing Covariates and in Complete Case Analyses

	**Ability to move around in home n (%)**		**Ability to engage in housework activity n (%)**		**Ability to engage in physical activity n (%)**		**Difficulty standing up after sitting in a chair n (%)**		**Difficulty walking alone up and down a flight of stairs n (%)**		**Difficulty walking 2-3 neighbourhood blocks n (%)**	
	**Not worse**	**Worse**	**Not worse**	**Worse**	**Not worse**	**Worse**	**Not worse**	**Worse**	**Not worse**	**Worse**	**Not worse**	**Worse**
**Missing covariates**												
Non-COVID-19	19274 (92.2)	1636 (7.8)	19320 (92.5)	1569 (7.5)	16009 (76.4)	4939 (23.6)	6955 (90.2)	753 (9.8)	7060 (91.8)	630 (8.2)	6897 (90.0)	767 (10.0)
Suspected COVID-19	1806 (81.8)	403 (18.2)	1822 (82.8)	378 (17.2)	1379 (62.2)	837 (37.8)	696 (83.6)	137 (16.4)	696 (83.4)	139 (16.6)	678 (81.2)	157 (18.8)
Probable/confirmed COVID-19	124 (86.1)	20 (13.9)	123 (85.4)	21 (14.6)	87 (60.0)	58 (40.0)	42 (82.4)	9 (17.6)	45 (88.2)	6 (11.8)	46 (90.2)	5 (9.8)
**Complete case analyses**												
Non-COVID-19	16965 (92.5)	1369 (7.5)	17011 (92.9)	1307 (7.1)	14086 (76.7)	4283 (23.3)	6297 (90.6)	657 (9.4)	6405 (92.3)	537 (7.7)	6263 (90.5)	656 (9.5)
Suspected COVID-19	1579 (81.9)	350 (18.1)	1603 (83.5)	317 (16.5)	1209 (62.5)	724 (37.5)	612 (83.3)	123 (16.7)	620 (83.9)	119 (16.1)	602 (81.5)	137 (18.5)
Probable/confirmed COVID-19	108 (87.8)	15 (12.2)	106 (86.2)	17 (13.8)	76 (61.3)	48 (38.7)	34 (81.0)	8 (19.0)	37 (88.1)	5 (11.9)	38 (90.5)	4 (9.5)

© 2022 Beauchamp MK et al. *JAMA Network Open.*

eTable 4. Descriptive Characteristics of Study Participants by COVID-19 Status in the CLSA COVID-19 Study

	**Participants at COVID-19 Baseline (n=28,559)**			**Participants at COVID-19 Exit (n=24,114)**		
	**Non-COVID**	**Suspected**	**Probable and confirmed**	**Non-COVID**	**Suspected**	**Probable and confirmed**
	**n=21275**	**n=2242**	**n=151**	**n=21275**	**n=2242**	**n=151**
**Age**						
<65	9166 (36.8)	961 (37.1)	64 (40.3)	7072 (33.2)	762 (34.0)	55 (36.4)
≥65	15774 (63.2)	1626 (62.9)	95 (59.7)	14203 (66.8)	1480 (66.0)	96 (63.6)
**Sex**						
Female	12966 (52.0)	1453 (56.2)	95 (59.7)	11200 (52.6)	1279 (57.0)	92 (60.9)
Male	11974 (48.0)	1134 (43.8)	64 (40.3)	10075 (47.4)	963 (43.0)	59 (39.1)
**Household income**						
Less than $50,000	5743 (24.5)	691 (28.5)	37 (25.9)	4936 (24.7)	603 (28.7)	35 (25.7)
$50,000 or more, but less than $100,000	8794 (37.6)	867 (35.8)	47 (32.9)	7615 (38.1)	760 (36.2)	44 (32.4)
$100,000 or more	8862 (37.9)	863 (35.6)	59 (41.3)	7429 (37.2)	738 (35.1)	57 (41.9)
**Dwelling type**						
House (single detached, semi-detached, duplex or townhouse	20122 (81.2)	2012 (78.3)	118 (75.2)	17170 (81.2)	1736 (77.9)	112 (75.2)
Apartment or condominium	4164 (16.8)	492 (19.1)	35 (22.3)	3575 (16.9)	433 (19.4)	33 (22.1)
Other	487 (2.0)	66 (2.6)	4 (2.5)	403 (1.9)	60 (2.7)	4 (2.7)
**Urban/Rural**						
Rural	3495 (14.1)	359 (14.0)	9 (5.7)	2937 (13.9)	305 (13.7)	9 (6.0)
Urban	21268	2208	148	18201	1921	140

© 2022 Beauchamp MK et al. *JAMA Network Open.*

	(85.9)	(86.0)	(94.3)	(86.1)	(86.3)	(94.0)
**Number of chronic conditions**						
Less than three	13569 (56.7)	1009 (40.3)	71 (46.4)	11491 (56.3)	857 (39.5)	67 (45.9)
Three or more	10356 (43.3)	1492 (59.7)	82 (53.6)	8927 (43.7)	1311 (60.5)	79 (54.1)
**Smoking**						
Former smoker	1471 (6.0)	186 (7.3)	6 (3.8)	12978 (61.7)	1442 (65.0)	110 (74.3)
Never smoker	15145 (61.5)	1659 (64.9)	113 (72.4)	6856 (32.6)	621 (28.0)	32 (21.6)
Current smoker	8013 (32.5)	710 (27.8)	37 (23.7)	1190 (5.7)	155 (7.0)	6 (4.1)
**Physical activity**						
Adequate activity	7987 (32.2)	683 (26.6)	55 (35.0)	6787 (32.1)	584 (26.2)	50 (33.6)
Low activity	16783 (67.8)	1886 (73.4)	102 (65.0)	14359 (67.9)	1644 (73.8)	99 (66.4)
**Nutritional risk**						
High risk	21009 (87.3)	1961 (79.1)	133 (87.5)	2497 (12.2)	435 (20.3)	19 (13.2)
Not at risk	3053 (12.7)	517 (20.9)	19 (12.5)	18041 (87.8)	1713 (79.7)	125 (86.8)

© 2022 Beauchamp MK et al. *JAMA Network Open.*

eFigure 2. Multivariable Analysis of the Association of COVID-19 With Worsening Mobility and Physical Function

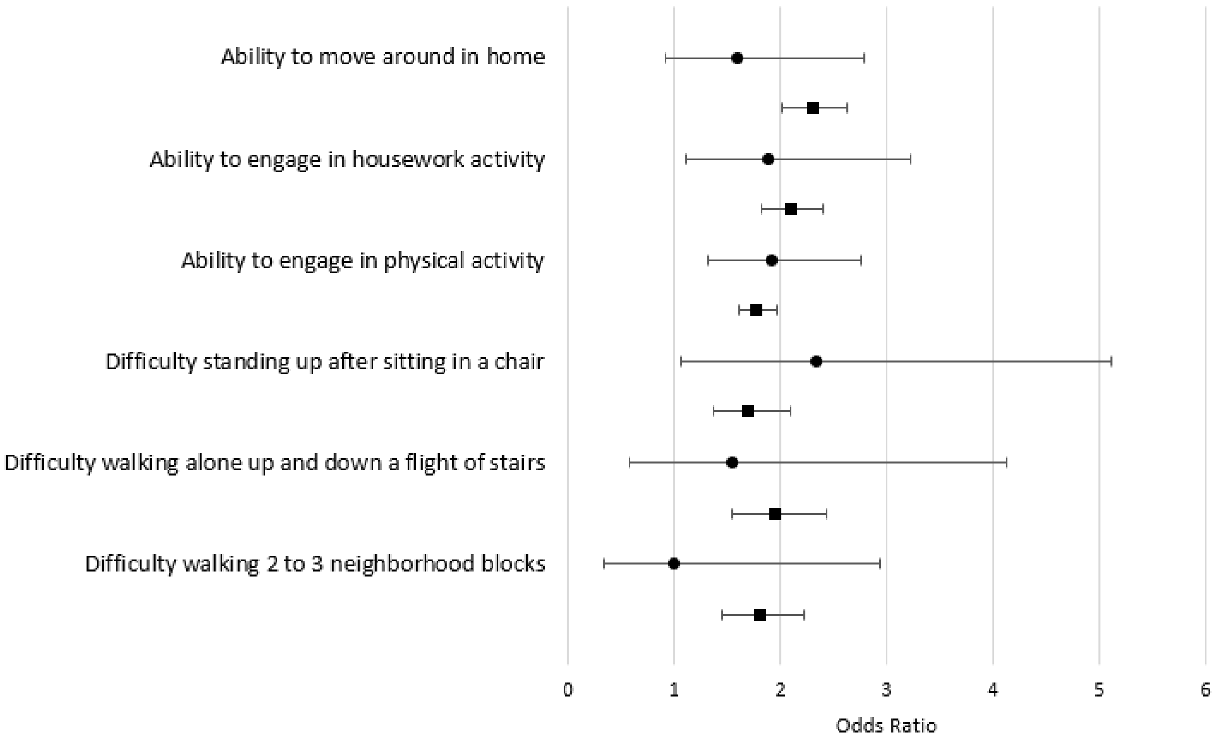

Note: Unadjusted odds ratio are presented with a circle and adjusted odds ratio are presented with a square. Models were adjusted for age, sex, annual household income, dwelling type, living area, number of chronic conditions, smoking status, physical activity, and nutritional risk

© 2022 Beauchamp MK et al. *JAMA Network Open.*

eTable 5. Multivariable Analysis of the Association of COVID-19 With Worsening of Mobility After Adjusting for Additional Covariates

	**Ability to move around in home (n=20,163)**	**Ability to engage in housework activity (n=20,137)**	**Ability to engage in physical activity (n=20,202)**
	**OR (95% CI)**	**OR (95% CI)**	**OR (95% CI)**
**Unadjusted model**			
**COVID-19 Status**			
Probable and confirmed COVID-19 vs. Non-COVID-19	1.90 (1.18, 3.06)	2.10 (1.32, 3.35)	2.16 (1.55, 3.02)
Suspected COVID-19 vs. Non-COVID-19	2.63 (2.33, 2.96)	2.56 (2.26, 2.89)	1.97 (1.80, 2.16)
**Adjusted model**			
**COVID-19 Status**			
Probable and confirmed COVID-19 vs. Non-COVID-19	1.61 (0.92, 2.81)	1.89 (1.11, 3.23)	1.95 (1.35, 2.82)
Suspected COVID-19 vs. Non-COVID-19	2.27 (1.98, 2.60)	2.06 (1.79, 2.37)	1.76 (1.59, 1.95)
**Age Group**			
≥65 vs. <65 years	1.48 (1.29, 1.68)	1.20 (1.06, 1.37)	1.05 (0.97, 1.13)
**Sex**			
Female vs. Male	1.03 (0.92, 1.14)	1.50 (1.34, 1.68)	1.19 (1.11, 1.27)
**Ethnicity**			
Non-European vs. European	0.99 (0.80, 1.22)	0.86 (0.69, 1.09)	1.04 (0.91, 1.19)
**Annual Household Income**			
<$50,000 vs. ≥$100,000	1.16 (1.00, 1.36)	1.43 (1.22, 1.67)	0.90 (0.81, 1.00)
≥$50,000 - <$100,000 vs. ≥$100,000	1.12 (0.98, 1.28)	1.20 (1.05, 1.38)	0.99 (0.91, 1.07)
**Social Participation**			
Low vs. High social participation	1.10 (0.97, 1.25)	1.27 (1.12, 1.44)	1.00 (0.91, 1.09)

© 2022 Beauchamp MK et al. *JAMA Network Open.*

**Number of People Living in the Same Household**			
Living alone vs. Not living alone	1.01 (0.88, 1.15)	1.07 (0.94, 1.22)	1.01 (0.92, 1.10)
**Dwelling Type**			
Apartment or condominium vs. House	1.20 (1.05, 1.37)	1.26 (1.10, 1.44)	1.24 (1.13, 1.35)
Other vs. House	1.01 (0.72, 1.40)	1.02 (0.73, 1.42)	1.04 (0.82, 1.33)
**Living Area**			
Urban vs. Rural	1.11 (0.95, 1.29)	1.15 (0.98, 1.35)	1.32 (1.19, 1.45)
**Number of Chronic Conditions**			
≥3 vs. <3	2.28 (2.03, 2.56)	2.18 (1.94, 2.46)	1.56 (1.46, 1.68)
**Type of Alcohol Drinker**			
Binge drinker vs. Non-drinker	1.12 (0.84, 1.49)	1.20 (0.90, 1.61)	1.04 (0.85, 1.26)
Regular drinker vs. Non-drinker	0.92 (0.79, 1.08)	1.00 (0.85, 1.17)	1.10 (0.99, 1.22)
Occasional drinker vs. Non-drinker	1.00 (0.82, 1.21)	0.98 (0.80, 1.19)	1.13 (0.99, 1.29)
**Smoking Status**			
Current smoker vs. Never smoker	1.22 (0.98, 1.52)	1.41 (1.13, 1.75)	0.99 (0.85, 1.15)
Former smoker vs. Never smoker	1.12 (1.00, 1.26)	1.15 (1.02, 1.30)	1.05 (0.98, 1.13)
**Physical Activity**			
Low activity vs. Adequate activity	1.25 (1.11, 1.41)	1.26 (1.11, 1.44)	0.91 (0.85, 0.98)
**Nutritional Risk**			
High risk vs. Not at risk	1.87 (1.64, 2.13)	2.13 (1.88, 2.43)	1.57 (1.43, 1.73)

*In addition to age, sex, annual household income, dwelling type, living area, number of chronic conditions, smoking status, physical activity, and nutritional risk, these models were further adjusted for ethnicity, social participation, number of people living in the same household, and alcohol consumption.

© 2022 Beauchamp MK et al. *JAMA Network Open.*

eTable 6. Multivariable Analysis of the Association of COVID-19 With Worsening Physical Function After Adjusting for Additional Covariates*

	**Difficulty standing up after sitting in a chair (n=7,633)**	**Difficulty walking alone up and down a flight of stairs (n=7,626)**	**Difficulty walking 2 to 3 neighborhood blocks (n=7,602)**
	**OR (95% CI)**	**OR (95% CI)**	**OR (95% CI)**
**Unadjusted model**			
**COVID-19 Status**			
Probable and confirmed COVID-19 vs. Non-COVID-19	1.98 (0.96, 4.08)	1.49 (0.64, 3.52)	0.98 (0.39, 2.47)
Suspected COVID-19 vs. Non-COVID-19	1.82 (1.49, 2.22)	2.24 (1.83, 2.73)	2.08 (1.72, 2.52)
**Adjusted model**			
**COVID-19 Status**			
Probable and confirmed COVID-19 vs. Non-COVID-19	2.37 (1.07, 5.22)	1.61 (0.60, 4.29)	1.04 (0.35, 3.03)
Suspected COVID-19 vs. Non-COVID-19	1.67 (1.34, 2.07)	1.88 (1.49, 2.36)	1.78 (1.43, 2.21)
**Age Group**			
≥65 vs. <65 years	1.29 (1.06, 1.55)	1.97 (1.56, 2.50)	1.59 (1.29, 1.96)
**Sex**			
Female vs. Male	0.93 (0.79, 1.09)	1.04 (0.87, 1.24)	0.97 (0.82, 1.14)
**Ethnicity**			
Non-European vs. European	0.80 (0.55, 1.14)	0.80 (0.54, 1.19)	0.68 (0.46, 0.99)
**Annual Household Income**			
<$50,000 vs. ≥$100,000	1.47 (1.17, 1.84)	1.72 (1.33, 2.22)	2.20 (1.71, 2.81)
≥$50,000 - <$100,000 vs. ≥$100,000	1.21 (0.99, 1.48)	1.32 (1.04, 1.68)	1.77 (1.41, 2.23)
**Social Participation**			
Low vs. High social participation	1.13 (0.95, 1.34)	1.12 (0.93, 1.36)	1.14 (0.95, 1.36)

© 2022 Beauchamp MK et al. *JAMA Network Open.*

**Number of People Living in the Same Household**			
Living alone vs. Not living alone	0.91 (0.75, 1.11)	1.05 (0.86, 1.29)	1.01 (0.84, 1.23)
**Dwelling Type**			
Apartment or condominium vs. House	1.01 (0.81, 1.26)	1.30 (1.04, 1.62)	1.09 (0.88, 1.36)
Other vs. House	0.90 (0.58, 1.38)	1.53 (1.04, 2.25)	1.30 (0.89, 1.89)
**Living Area**			
Urban vs. Rural	0.96 (0.80, 1.14)	1.05 (0.86, 1.28)	0.97 (0.81, 1.17)
**Number of Chronic Conditions**			
≥3 vs. <3	1.74 (1.48, 2.06)	2.38 (1.96, 2.88)	2.90 (2.42, 3.47)
**Type of Alcohol Drinker**			
Binge drinker vs. Non-drinker	0.91 (0.60, 1.38)	1.02 (0.66, 1.59)	0.56 (0.35, 0.88)
Regular drinker vs. Non-drinker	0.90 (0.72, 1.13)	0.77 (0.61, 0.97)	0.64 (0.52, 0.79)
Occasional drinker vs. Non-drinker	0.96 (0.73, 1.27)	0.95 (0.71, 1.26)	0.66 (0.50, 0.86)
**Smoking Status**			
Current smoker vs. Never smoker	1.07 (0.76, 1.49)	1.09 (0.76, 1.57)	1.22 (0.88, 1.69)
Former smoker vs. Never smoker	1.19 (1.00, 1.42)	1.24 (1.02, 1.51)	1.19 (0.99, 1.42)
**Physical Activity**			
Low activity vs. Adequate activity	1.24 (1.03, 1.50)	1.49 (1.20, 1.86)	1.50 (1.22, 1.83)
**Nutritional Risk**			
High risk vs. Not at risk	1.51 (1.23, 1.86)	1.60 (1.28, 1.98)	1.78 (1.46, 2.17)

*In addition to age, sex, annual household income, dwelling type, living area, number of chronic conditions, smoking status, physical activity, and nutritional risk, these models were further adjusted for ethnicity, social participation, number of people living in the same household, and alcohol consumption.

© 2022 Beauchamp MK et al. *JAMA Network Open.*

eTable 7. Multivariable Logistic Regression Models of the Association Between COVID-19 and Worsening Ability to Move Around in Home Since the Start of the Pandemic

	**Main effects**	**Interaction between COVID-19 Status and Age group**	**Interaction between COVID-19 Status and Sex**	**Interaction between COVID-19 Status and Household Income**	**Interaction between COVID-19 Status and Number of Chronic Conditions**
	**OR (95% CI)**	**OR (95% CI)**	**OR (95% CI)**	**OR (95% CI)**	**OR (95% CI)**
**COVID-19 Status**					
**Non-COVID-19 (reference)**					
Probable or confirmed COVID-19 vs. Non-COVID-19	1.60 (0.92, 2.79)	1.98 (0.69, 5.68)	1.28 (0.45, 3.62)	1.58 (0.61, 4.07)	0.74 (0.18, 3.04)
Suspected COVID-19 vs. Non-COVID-19	2.30 (2.01, 2.63)	2.18 (1.67, 2.86)	2.71 (2.23, 3.31)	2.40 (1.88, 3.07)	2.65 (2.08, 3.39)
**Age Group**					
≥65 vs. <65 years	1.47 (1.29, 1.67)	1.45 (1.26, 1.68)	1.47 (1.29, 1.67)	1.47 (1.29, 1.67)	1.47 (1.29, 1.68)
**Sex**					
Female vs. Male	1.02 (0.92, 1.13)	1.02 (0.92, 1.13)	1.07 (0.95, 1.20)	1.02 (0.92, 1.13)	1.02 (0.92, 1.13)
**Annual Household Income**					
<$50,000 vs. ≥$100,000	1.19 (1.03, 1.38)	1.19 (1.03, 1.38)	1.19 (1.03, 1.38)	1.19 (1.01, 1.39)	1.19 (1.03, 1.38)
≥$50,000 - <$100,000 vs. ≥$100,000	1.14 (1.00, 1.30)	1.14 (1.00, 1.29)	1.14 (1.00, 1.29)	1.17 (1.01, 1.35)	1.14 (1.00, 1.29)
**Dwelling Type**					
Apartment or condominium vs. House	1.20 (1.05, 1.36)	1.19 (1.05, 1.36)	1.19 (1.05, 1.36)	1.19 (1.05, 1.36)	1.20 (1.05, 1.36)
Other vs. House	1.05 (0.76, 1.45)	1.05 (0.76, 1.45)	1.05 (0.76, 1.46)	1.05 (0.76, 1.45)	1.05 (0.76, 1.45)
**Living Area**					
Urban vs. Rural	1.10 (0.95, 1.28)	1.10 (0.95, 1.28)	1.11 (0.95, 1.29)	1.10 (0.95, 1.29)	1.10 (0.95, 1.29)
**Number of Chronic Conditions**					
≥3 vs. <3	2.27 (2.03, 2.55)	2.27 (2.03, 2.55)	2.27 (2.03, 2.55)	2.27 (2.02, 2.54)	2.33 (2.06, 2.63)
**Smoking Status**					
Current smoker vs. Never smoker	1.27 (1.02, 1.58)	1.27 (1.02, 1.58)	1.27 (1.02, 1.58)	1.27 (1.02, 1.58)	1.27 (1.02, 1.58)

© 2022 Beauchamp MK et al. *JAMA Network Open.*

Former smoker vs. Never smoker	1.12 (1.00, 1.26)	1.12 (1.00, 1.26)	1.12 (1.00, 1.26)	1.12 (1.00, 1.26)	1.12 (1.00, 1.26)
**Physical Activity**					
Low activity vs. Adequate activity	1.27 (1.13, 1.44)	1.27 (1.13, 1.44)	1.27 (1.13, 1.44)	1.27 (1.13, 1.44)	1.27 (1.13, 1.43)
**Nutritional Risk**					
High risk vs. Not at risk	1.88 (1.66, 2.14)	1.88 (1.66, 2.14)	1.88 (1.66, 2.13)	1.88 (1.66, 2.14)	1.89 (1.66, 2.14)
**COVID-19 Status (ref = Non-COVID-19) by Age Group (ref = less than 65)**					
'Probable/Confirmed COVID-19' vs. 'Non-COVID-19' at '≥65' vs. '<65 years'		2.16 (1.12, 4.18)			
'Suspected COVID-19' vs. 'Non-COVID-19' at '≥65' vs. '<65 years'		3.40 (2.81, 4.11)			
**COVID-19 Status (ref = Non-COVID-19) by Sex (ref = Male)**					
'Probable/Confirmed COVID-19' vs. 'Non-COVID-19' at 'Female' vs. 'Male'			1.88 (0.97, 3.66)		
'Suspected COVID-19' vs. 'Non-COVID-19' at 'Female' vs. 'Male'			2.17 (1.80, 2.61)		
**COVID-19 Status (ref = Non-COVID-19) by Income (ref = ≥$100,000)**					
'Probable/Confirmed COVID-19' vs. 'Non-COVID-19' at '<$50,000' vs. '≥$100,000'				2.38 (0.95, 5.96)	
'Probable/Confirmed COVID-19' vs. 'Non-COVID-19' at '≥$50,000 - <$100,000' vs. '≥$100,000'				1.48 (0.52, 4.26)	
'Suspected COVID-19' vs. 'Non-COVID-19' at '<$50,000' vs. '≥$100,000'				2.93 (2.30, 3.72)	
'Suspected COVID-19' vs. 'Non-COVID-19' at '≥$50,000 - <$100,000' vs. '≥$100,000'				2.43 (1.93, 3.07)	
**COVID-19 Status (Non-COVID-19) by Number of Chronic Conditions (ref = <3)**					
'Probable/Confirmed COVID-19' vs. 'Non-COVID-19' at 'Female' vs. 'Male'					4.68 (2.50, 8.75)
'Suspected COVID-19' vs. 'Non-COVID-19' at 'Female' vs. 'Male'					5.07 (4.26, 6.03)

© 2022 Beauchamp MK et al. *JAMA Network Open.*

© 2022 Beauchamp MK et al. *JAMA Network Open.*

eTable 8. Multivariable Logistic Regression Models of the Association Between COVID-19 and Worsening Ability to Engage in Household Activity Since the Start of the Pandemic

	**Main effects**	**Interaction between COVID-19 Status and Age group**	**Interaction between COVID-19 Status and Sex**	**Interaction between COVID-19 Status and Household Income**	**Interaction between COVID-19 Status and Number of Chronic Conditions**
	**OR (95% CI)**	**OR (95% CI)**	**OR (95% CI)**	**OR (95% CI)**	**OR (95% CI)**
**COVID-19 Status**					
**Non-COVID-19 (reference)**					
Probable or confirmed COVID-19 vs. Non-COVID-19	1.89 (1.11, 3.22)	2.95 (1.21, 7.24)	2.24 (0.87, 5.77)	2.21 (0.91, 5.35)	1.14 (0.35, 3.69)
Suspected COVID-19 vs. Non-COVID-19	2.09 (1.82, 2.41)	2.15 (1.65, 2.80)	2.40 (1.91, 3.00)	2.10 (1.61, 2.75)	2.40 (1.86, 3.11)
**Age Group**					
≥65 vs. <65 years	1.19 (1.04, 1.35)	1.20 (1.04, 1.39)	1.19 (1.04, 1.35)	1.19 (1.04, 1.35)	1.19 (1.04, 1.35)
**Sex**					
Female vs. Male	1.50 (1.34, 1.67)	1.50 (1.34, 1.67)	1.55 (1.38, 1.76)	1.50 (1.34, 1.67)	1.50 (1.34, 1.67)
**Annual Household Income**					
<$50,000 vs. ≥$100,000	1.50 (1.29, 1.74)	1.50 (1.29, 1.74)	1.50 (1.29, 1.74)	1.50 (1.27, 1.76)	1.50 (1.29, 1.74)
≥$50,000 - <$100,000 vs. ≥$100,000	1.22 (1.06, 1.40)	1.22 (1.06, 1.40)	1.22 (1.06, 1.40)	1.23 (1.06, 1.44)	1.22 (1.06, 1.40)
**Dwelling Type**					
Apartment or condominium vs. House	1.27 (1.12, 1.44)	1.27 (1.12, 1.44)	1.27 (1.12, 1.44)	1.27 (1.12, 1.44)	1.27 (1.12, 1.44)
Other vs. House	1.06 (0.77, 1.47)	1.06 (0.77, 1.47)	1.06 (0.77, 1.47)	1.06 (0.77, 1.47)	1.06 (0.77, 1.47)
**Living Area**					
Urban vs. Rural	1.16 (0.99, 1.36)	1.16 (0.99, 1.36)	1.16 (0.99, 1.36)	1.16 (0.99, 1.36)	1.16 (0.99, 1.36)
**Number of Chronic Conditions**					
≥3 vs. <3	2.16 (1.92, 2.43)	2.15 (1.91, 2.42)	2.15 (1.92, 2.42)	2.15 (1.91, 2.42)	2.20 (1.94, 2.50)
**Smoking Status**					
Current smoker vs. Never smoker	1.48 (1.20, 1.83)	1.48 (1.20, 1.83)	1.48 (1.20, 1.83)	1.48 (1.20, 1.83)	1.48 (1.20, 1.83)

© 2022 Beauchamp MK et al. *JAMA Network Open.*

Former smoker vs. Never smoker	1.16 (1.03, 1.31)	1.16 (1.03, 1.31)	1.16 (1.03, 1.30)	1.16 (1.03, 1.31)	1.16 (1.03, 1.31)
**Physical Activity**					
Low activity vs. Adequate activity	1.30 (1.15, 1.48)	1.31 (1.15, 1.48)	1.30 (1.15, 1.48)	1.30 (1.15, 1.48)	1.30 (1.15, 1.48)
**Nutritional Risk**					
High risk vs. Not at risk	2.20 (1.94, 2.50)	2.20 (1.94, 2.50)	2.20 (1.94, 2.49)	2.20 (1.94, 2.50)	2.20 (1.95, 2.50)
**COVID-19 Status (ref = Non-COVID-19) by Age Group (ref = less than 65)**					
'Probable/Confirmed COVID-19' vs. 'Non-COVID-19' at '≥65' vs. '<65 years'		1.87 (0.96, 3.65)			
'Suspected COVID-19' vs. 'Non-COVID-19' at '≥65' vs. '<65 years'		2.50 (2.05, 3.04)			
**COVID-19 Status (ref = Non-COVID-19) by Sex (ref = Male)**					
'Probable/Confirmed COVID-19' vs. 'Non-COVID-19' at 'Female' vs. 'Male'			2.73 (1.43, 5.20)		
'Suspected COVID-19' vs. 'Non-COVID-19' at 'Female' vs. 'Male'			3.01 (2.50, 3.64)		
**COVID-19 Status (ref = Non-COVID-19) by Income (ref = ≥$100,000)**					
'Probable/Confirmed COVID-19' vs. 'Non-COVID-19' at '<$50,000' vs. '≥$100,000'				3.21 (1.33, 7.74)	
'Probable/Confirmed COVID-19' vs. 'Non-COVID-19' at '≥$50,000 - <$100,000' vs. '≥$100,000'				1.65 (0.57, 4.77)	
'Suspected COVID-19' vs. 'Non-COVID-19' at '<$50,000' vs. '≥$100,000'				3.21 (2.51, 4.11)	
'Suspected COVID-19' vs. 'Non-COVID-19' at '≥$50,000 - <$100,000' vs. '≥$100,000'				2.51 (1.97, 3.20)	
**COVID-19 Status (Non-COVID-19) by Number of Chronic Conditions (ref = <3)**					
'Probable/Confirmed COVID-19' vs. 'Non-COVID-19' at 'Female' vs. 'Male'					4.98 (2.68, 9.24)
'Suspected COVID-19' vs. 'Non-COVID-19' at 'Female' vs. 'Male'					4.38 (3.66, 5.25)

© 2022 Beauchamp MK et al. *JAMA Network Open.*

eTable 9. Multivariable Logistic Regression Models of the Association Between COVID-19 and Worsening Ability to Engage in Physical Activity Since the Start of the Pandemic

	Main effects	Interaction between COVID-19 Status and Age group	Interaction between COVID-19 Status and Sex	Interaction between COVID-19 Status and Household Income	Interaction between COVID-19 Status and Number of Chronic Conditions
	**OR (95% CI)**	**OR (95% CI)**	**OR (95% CI)**	**OR (95% CI)**	**OR (95% CI)**
**COVID-19 Status**					
**Non-COVID-19 (reference)**					
Probable or confirmed COVID-19 vs. Non-COVID-19	1.91 (1.32, 2.76)	2.03 (1.10, 3.77)	2.11 (1.18, 3.79)	2.14 (1.23, 3.72)	1.90 (1.11, 3.26)
Suspected COVID-19 vs. Non-COVID-19	1.78 (1.61, 1.97)	1.51 (1.27, 1.80)	2.05 (1.76, 2.38)	1.60 (1.35, 1.89)	1.70 (1.45, 2.00)
**Age Group**					
≥65 vs. <65 years	1.05 (0.97, 1.13)	1.02 (0.94, 1.11)	1.05 (0.97, 1.13)	1.05 (0.97, 1.13)	1.05 (0.97, 1.13)
**Sex**					
Female vs. Male	1.20 (1.12, 1.28)	1.20 (1.12, 1.28)	1.23 (1.15, 1.32)	1.20 (1.12, 1.28)	1.19 (1.12, 1.28)
**Annual Household Income**					
<$50,000 vs. ≥$100,000	0.90 (0.82, 0.99)	0.90 (0.82, 0.99)	0.90 (0.82, 0.99)	0.87 (0.79, 0.96)	0.90 (0.82, 0.99)
≥$50,000 - <$100,000 vs. ≥$100,000	0.99 (0.92, 1.07)	0.99 (0.92, 1.07)	0.99 (0.91, 1.07)	0.98 (0.90, 1.07)	0.99 (0.92, 1.07)
**Dwelling Type**					
Apartment or condominium vs. House	1.24 (1.13, 1.35)	1.23 (1.13, 1.34)	1.24 (1.13, 1.35)	1.24 (1.13, 1.35)	1.24 (1.13, 1.35)
Other vs. House	1.06 (0.84, 1.34)	1.06 (0.84, 1.34)	1.06 (0.83, 1.34)	1.06 (0.83, 1.34)	1.06 (0.84, 1.34)
**Living Area**					
Urban vs. Rural	1.33 (1.20, 1.47)	1.33 (1.20, 1.47)	1.33 (1.20, 1.47)	1.33 (1.20, 1.47)	1.33 (1.20, 1.47)
**Number of Chronic Conditions**					
≥3 vs. <3	1.55 (1.45, 1.67)	1.55 (1.45, 1.67)	1.55 (1.45, 1.67)	1.55 (1.45, 1.67)	1.54 (1.43, 1.66)
**Smoking Status**					
Current smoker vs. Never smoker	0.99 (0.86, 1.15)	0.99 (0.86, 1.15)	0.99 (0.86, 1.15)	0.99 (0.86, 1.15)	0.99 (0.86, 1.15)

© 2022 Beauchamp MK et al. *JAMA Network Open.*

Former smoker vs. Never smoker	1.05 (0.98, 1.12)	1.05 (0.97, 1.12)	1.05 (0.97, 1.12)	1.05 (0.97, 1.12)	1.05 (0.98, 1.12)
**Physical Activity**					
Low activity vs. Adequate activity	0.91 (0.85, 0.98)	0.91 (0.85, 0.98)	0.91 (0.85, 0.98)	0.91 (0.85, 0.98)	0.91 (0.85, 0.98)
**Nutritional Risk**					
High risk vs. Not at risk	1.58 (1.44, 1.73)	1.57 (1.44, 1.73)	1.58 (1.44, 1.73)	1.57 (1.43, 1.73)	1.57 (1.44, 1.73)
**COVID-19 Status (ref = Non-COVID-19) by Age Group (ref = less than 65)**					
‘Probable/Confirmed COVID-19’ vs. ‘Non-COVID-19’ at ‘≥65’ vs. ‘<65 years’		1.88 (1.18, 2.98)			
‘Suspected COVID-19’ vs. ‘Non-COVID-19’ at ‘≥65’ vs. ‘<65 years’		1.97 (1.72, 2.25)			
**COVID-19 Status (ref = Non-COVID-19) by Sex (ref = Male)**					
‘Probable/Confirmed COVID-19’ vs. ‘Non-COVID-19’ at ‘Female’ vs. ‘Male’			2.20 (1.37, 3.52)		
‘Suspected COVID-19’ vs. ‘Non-COVID-19’ at ‘Female’ vs. ‘Male’			1.97 (1.71, 2.25)		
**COVID-19 Status (ref = Non-COVID-19) by Income (ref = ≥$100,000)**					
‘Probable/Confirmed COVID-19’ vs. ‘Non-COVID-19’ at ‘<$50,000’ vs. ‘≥$100,000’				1.64 (0.78, 3.45)	
‘Probable/Confirmed COVID-19’ vs. ‘Non-COVID-19’ at ‘≥$50,000 - <$100,000’ vs. ‘≥$100,000’				1.61 (0.83, 3.12)	
‘Suspected COVID-19’ vs. ‘Non-COVID-19’ at ‘<$50,000’ vs. ‘≥$100,000’				1.80 (1.49, 2.18)	
‘Suspected COVID-19’ vs. ‘Non-COVID-19’ at ‘≥$50,000 - <$100,000’ vs. ‘≥$100,000’				1.73 (1.46, 2.04)	
**COVID-19 Status (Non-COVID-19) by Number of Chronic Conditions (ref = <3)**					
‘Probable/Confirmed COVID-19’ vs. ‘Non-COVID-19’ at ‘Female’ vs. ‘Male’					2.95 (1.78, 4.88)
‘Suspected COVID-19’ vs. ‘Non-COVID-19’ at ‘Female’ vs. ‘Male’					2.82 (2.47, 3.21)

© 2022 Beauchamp MK et al. *JAMA Network Open.*

eTable 10. Multivariable Logistic Regression Models of the Association Between COVID-19 and Worsening Difficulty Standing Up After Sitting in a Chair

	**Main effects**	**Interaction between COVID-19 Status and Age group**	**Interaction between COVID-19 Status and Sex**	**Interaction between COVID-19 Status and Household Income**	**Interaction between COVID-19 Status and Number of Chronic Conditions**
	**OR (95% CI)**	**OR (95% CI)**	**OR (95% CI)**	**OR (95% CI)**	**OR (95% CI)**
**COVID-19 Status**					
**Non-COVID-19 (reference)**					
Probable or confirmed COVID-19 vs. Non-COVID-19	2.33 (1.06, 5.11)	2.34 (0.51, 10.71)	2.49 (0.83, 7.47)	2.09 (0.47, 9.38)	2.71 (0.91, 8.07)
Suspected COVID-19 vs. Non-COVID-19	1.70 (1.37, 2.10)	1.74 (1.16, 2.59)	1.76 (1.27, 2.43)	1.82 (1.20, 2.76)	1.99 (1.37, 2.87)
**Age Group**					
≥65 vs. <65 years	1.26 (1.05, 1.52)	1.27 (1.04, 1.55)	1.26 (1.05, 1.52)	1.26 (1.05, 1.52)	1.26 (1.05, 1.52)
**Sex**					
Female vs. Male	0.93 (0.79, 1.08)	0.92 (0.79, 1.08)	0.93 (0.79, 1.08)	0.92 (0.79, 1.08)	0.92 (0.79, 1.08)
**Annual Household Income**					
<$50,000 vs. ≥$100,000	1.48 (1.19, 1.83)	1.48 (1.19, 1.83)	1.48 (1.19, 1.83)	1.48 (1.18, 1.87)	1.47 (1.19, 1.83)
≥$50,000 - <$100,000 vs. ≥$100,000	1.22 (1.00, 1.49)	1.22 (1.00, 1.49)	1.22 (1.00, 1.49)	1.25 (1.01, 1.55)	1.22 (1.00, 1.48)
**Dwelling Type**					
Apartment or condominium vs. House	0.96 (0.78, 1.19)	0.96 (0.78, 1.19)	0.96 (0.78, 1.19)	0.96 (0.78, 1.19)	0.96 (0.78, 1.19)
Other vs. House	0.87 (0.57, 1.34)	0.87 (0.57, 1.34)	0.87 (0.56, 1.34)	0.87 (0.57, 1.34)	0.87 (0.57, 1.34)
**Living Area**					
Urban vs. Rural	0.96 (0.80, 1.14)	0.96 (0.80, 1.14)	0.96 (0.80, 1.14)	0.95 (0.80, 1.14)	0.96 (0.80, 1.14)
**Number of Chronic Conditions**					
≥3 vs. <3	1.75 (1.49, 2.06)	1.75 (1.49, 2.06)	1.75 (1.49, 2.06)	1.75 (1.48, 2.06)	1.81 (1.52, 2.15)
**Smoking Status**					
Current smoker vs. Never smoker	1.08 (0.78, 1.50)	1.08 (0.78, 1.49)	1.08 (0.78, 1.49)	1.08 (0.78, 1.50)	1.07 (0.77, 1.49)

© 2022 Beauchamp MK et al. *JAMA Network Open.*

Former smoker vs. Never smoker	1.16 (0.98, 1.38)	1.16 (0.98, 1.38)	1.16 (0.98, 1.38)	1.16 (0.98, 1.38)	1.16 (0.98, 1.38)
**Physical Activity**					
Low activity vs. Adequate activity	1.29 (1.07, 1.55)	1.29 (1.07, 1.55)	1.29 (1.07, 1.55)	1.29 (1.07, 1.55)	1.29 (1.07, 1.55)
**Nutritional Risk**					
High risk vs. Not at risk	1.52 (1.24, 1.86)	1.52 (1.24, 1.85)	1.52 (1.24, 1.86)	1.51 (1.24, 1.85)	1.52 (1.24, 1.86)
**COVID-19 Status (ref = Non-COVID-19) by Age Group (ref = less than 65)**					
‘Probable/Confirmed COVID-19’ vs. ‘Non-COVID-19’ at ‘≥65’ vs. ‘<65 years’		2.94 (1.15, 7.47)			
‘Suspected COVID-19’ vs. ‘Non-COVID-19’ at ‘≥65’ vs. ‘<65 years’		2.13 (1.58, 2.87)			
**COVID-19 Status (ref = Non-COVID-19) by Sex (ref = Male)**					
‘Probable/Confirmed COVID-19’ vs. ‘Non-COVID-19’ at ‘Female’ vs. ‘Male’			2.03 (0.66, 6.28)		
‘Suspected COVID-19’ vs. ‘Non-COVID-19’ at ‘Female’ vs. ‘Male’			1.54 (1.15, 2.07)		
**COVID-19 Status (ref = Non-COVID-19) by Income (ref = ≥$100,000)**					
‘Probable/Confirmed COVID-19’ vs. ‘Non-COVID-19’ at ‘<$50,000’ vs. ‘≥$100,000’				7.06 (2.18, 22.92)	
‘Probable/Confirmed COVID-19’ vs. ‘Non-COVID-19’ at ‘≥$50,000 - <$100,000’ vs. ‘≥$100,000’				0.94 (0.12, 7.27)	
‘Suspected COVID-19’ vs. ‘Non-COVID-19’ at ‘<$50,000’ vs. ‘≥$100,000’				2.51 (1.74, 3.63)	
‘Suspected COVID-19’ vs. ‘Non-COVID-19’ at ‘≥$50,000 - <$100,000’ vs. ‘≥$100,000’				2.01 (1.37, 2.95)	
**COVID-19 Status (Non-COVID-19) by Number of Chronic Conditions (ref = <3)**					
‘Probable/Confirmed COVID-19’ vs. ‘Non-COVID-19’ at ‘Female’ vs. ‘Male’					3.62 (1.17, 11.22)
‘Suspected COVID-19’ vs. ‘Non-COVID-19’ at ‘Female’ vs. ‘Male’					2.85 (2.16, 3.75)

© 2022 Beauchamp MK et al. *JAMA Network Open.*

© 2022 Beauchamp MK et al. *JAMA Network Open.*

eTable 11. Multivariable Logistic Regression Models of the Association Between COVID-19 and Worsening Difficulty Walking Alone Up and Down a Flight of Stairs

	**Main effects**	**Interaction between COVID-19 Status and Age group**	**Interaction between COVID-19 Status and Sex**	**Interaction between COVID-19 Status and Household Income**
	**OR (95% CI)**	**OR (95% CI)**	**OR (95% CI)**	**OR (95% CI)**
**COVID-19 Status**				
**Non-COVID-19 (reference)**				
Probable or confirmed COVID-19 vs. Non-COVID-19	1.55 (0.58, 4.13)	2.10 (0.26, 16.64)	2.56 (0.71, 9.28)	2.00 (0.26, 15.54)
Suspected COVID-19 vs. Non-COVID-19	1.95 (1.55, 2.44)	1.33 (0.76, 2.33)	2.44 (1.74, 3.43)	1.83 (1.11, 3.02)
**Age Group**				
≥65 vs. <65 years	1.96 (1.55, 2.48)	1.82 (1.41, 2.34)	1.96 (1.55, 2.48)	1.97 (1.56, 2.49)
**Sex**				
Female vs. Male	1.01 (0.85, 1.20)	1.01 (0.85, 1.20)	1.09 (0.90, 1.31)	1.01 (0.85, 1.20)
**Annual Household Income**				
<$50,000 vs. ≥$100,000	1.84 (1.44, 2.35)	1.84 (1.44, 2.35)	1.84 (1.44, 2.36)	1.87 (1.43, 2.44)
≥$50,000 - <$100,000 vs. ≥$100,000	1.37 (1.08, 1.74)	1.37 (1.08, 1.74)	1.37 (1.08, 1.74)	1.31 (1.01, 1.70)
**Dwelling Type**				
Apartment or condominium vs. House	1.33 (1.07, 1.64)	1.32 (1.07, 1.63)	1.32 (1.07, 1.64)	1.33 (1.08, 1.65)
Other vs. House	1.61 (1.10, 2.36)	1.62 (1.10, 2.36)	1.60 (1.10, 2.35)	1.62 (1.11, 2.37)
**Living Area**				
Urban vs. Rural	1.03 (0.85, 1.26)	1.03 (0.85, 1.26)	1.03 (0.85, 1.26)	1.03 (0.84, 1.25)
**Number of Chronic Conditions**				
≥3 vs. <3	2.38 (1.97, 2.87)	2.38 (1.97, 2.87)	2.38 (1.97, 2.87)	2.38 (1.97, 2.87)
**Smoking Status**				
Current smoker vs. Never smoker	1.11 (0.78, 1.58)	1.11 (0.78, 1.58)	1.11 (0.78, 1.58)	1.11 (0.78, 1.58)

© 2022 Beauchamp MK et al. *JAMA Network Open.*

Former smoker vs. Never smoker	1.20 (0.99, 1.45)	1.20 (0.99, 1.45)	1.20 (0.99, 1.44)	1.20 (0.99, 1.45)
**Physical Activity**				
Low activity vs. Adequate activity	1.56 (1.25, 1.94)	1.56 (1.26, 1.95)	1.57 (1.26, 1.95)	1.56 (1.25, 1.94)
**Nutritional Risk**				
High risk vs. Not at risk	1.60 (1.29, 1.97)	1.60 (1.29, 1.98)	1.59 (1.29, 1.97)	1.60 (1.29, 1.97)
**COVID-19 Status (ref = Non-COVID-19) by Age Group (ref = less than 65)**				
'Probable/Confirmed COVID-19' vs. 'Non-COVID-19' at '≥65' vs. '<65 years'		2.61 (0.85, 8.02)		
'Suspected COVID-19' vs. 'Non-COVID-19' at '≥65' vs. '<65 years'		3.84 (2.77, 5.32)		
**COVID-19 Status (ref = Non-COVID-19) by Sex (ref = Male)**				
'Probable/Confirmed COVID-19' vs. 'Non-COVID-19' at 'Female' vs. 'Male'			1.02 (0.23, 4.63)	
'Suspected COVID-19' vs. 'Non-COVID-19' at 'Female' vs. 'Male'			1.79 (1.30, 2.45)	
**COVID-19 Status (ref = Non-COVID-19) by Income (ref = ≥$100,000)**				
'Probable/Confirmed COVID-19' vs. 'Non-COVID-19' at '<$50,000' vs. '≥$100,000'				3.51 (0.90, 13.75)
'Probable/Confirmed COVID-19' vs. 'Non-COVID-19' at '≥$50,000 - <$100,000' vs. '≥$100,000'				1.17 (0.15, 9.35)
'Suspected COVID-19' vs. 'Non-COVID-19' at '<$50,000' vs. '≥$100,000'				3.04 (2.05, 4.51)
'Suspected COVID-19' vs. 'Non-COVID-19' at '≥$50,000 - <$100,000' vs. '≥$100,000'				3.26 (2.19, 4.84)
**COVID-19 Status (Non-COVID-19) by Number of Chronic Conditions (ref = <3)**				
'Probable/Confirmed COVID-19' vs. 'Non-COVID-19' at 'Female' vs. 'Male'				
'Suspected COVID-19' vs. 'Non-COVID-19' at 'Female' vs. 'Male'				

© 2022 Beauchamp MK et al. *JAMA Network Open.*

eTable 12. Multivariable Logistic Regression Models of the Association Between COVID-19 and Worsening Difficulty Walking 2 to 3 Neighborhood Blocks

	**Main effects**	**Interaction between COVID-19 Status and Age group**	**Interaction between COVID-19 Status and Sex**	**Interaction between COVID-19 Status and Number of Chronic Conditions**
	**OR (95% CI)**	**OR (95% CI)**	**OR (95% CI)**	**OR (95% CI)**
**COVID-19 Status**				
**Non-COVID-19 (reference)**				
Probable or confirmed COVID-19 vs. Non-COVID-19	1.00 (0.34, 2.93)	1.74 (0.22, 13.82)	1.42 (0.31, 6.47)	0.85 (0.11, 6.46)
Suspected COVID-19 vs. Non-COVID-19	1.80 (1.45, 2.22)	1.62 (1.02, 2.57)	2.49 (1.81, 3.42)	1.95 (1.25, 3.03)
**Age Group**				
≥65 vs. <65 years	1.56 (1.27, 1.92)	1.53 (1.23, 1.92)	1.56 (1.27, 1.92)	1.57 (1.27, 1.93)
**Sex**				
Female vs. Male	0.98 (0.83, 1.15)	0.98 (0.83, 1.15)	1.07 (0.90, 1.28)	0.98 (0.83, 1.15)
**Annual Household Income**				
<\$50,000 vs. ≥\$100,000	2.35 (1.86, 2.98)	2.35 (1.86, 2.98)	2.35 (1.86, 2.98)	2.35 (1.86, 2.97)
≥\$50,000 - <\$100,000 vs. ≥\$100,000	1.77 (1.41, 2.22)	1.77 (1.41, 2.23)	1.77 (1.41, 2.22)	1.77 (1.41, 2.22)
**Dwelling Type**				
Apartment or condominium vs. House	1.07 (0.87, 1.32)	1.07 (0.87, 1.31)	1.07 (0.87, 1.31)	1.07 (0.87, 1.31)
Other vs. House	1.28 (0.88, 1.85)	1.28 (0.88, 1.85)	1.27 (0.88, 1.85)	1.28 (0.88, 1.85)
**Living Area**				
Urban vs. Rural	0.96 (0.80, 1.15)	0.96 (0.80, 1.15)	0.96 (0.80, 1.15)	0.96 (0.80, 1.15)
**Number of Chronic Conditions**				
≥3 vs. <3	3.01 (2.52, 3.59)	3.01 (2.52, 3.60)	3.01 (2.52, 3.59)	3.05 (2.52, 3.68)
**Smoking Status**				
Current smoker vs. Never smoker	1.20 (0.88, 1.66)	1.20 (0.88, 1.65)	1.20 (0.88, 1.65)	1.20 (0.87, 1.65)

© 2022 Beauchamp MK et al. *JAMA Network Open.*

Former smoker vs. Never smoker	1.11 (0.93, 1.32)	1.11 (0.93, 1.32)	1.10 (0.93, 1.32)	1.11 (0.93, 1.32)
**Physical Activity**				
Low activity vs. Adequate activity	1.55 (1.26, 1.89)	1.55 (1.27, 1.90)	1.56 (1.27, 1.91)	1.55 (1.26, 1.89)
**Nutritional Risk**				
High risk vs. Not at risk	1.83 (1.51, 2.22)	1.83 (1.51, 2.22)	1.82 (1.50, 2.21)	1.83 (1.51, 2.22)
**COVID-19 Status (ref = Non-COVID-19) by Age Group (ref = less than 65)**				
'Probable/Confirmed COVID-19' vs. 'Non-COVID-19' at '≥65' vs. '<65 years'		1.33 (0.38, 4.63)		
'Suspected COVID-19' vs. 'Non-COVID-19' at '≥65' vs. '<65 years'		2.84 (2.09, 3.84)		
**COVID-19 Status (ref = Non-COVID-19) by Sex (ref = Male)**				
'Probable/Confirmed COVID-19' vs. 'Non-COVID-19' at 'Female' vs. 'Male'			0.81 (0.18, 3.68)	
'Suspected COVID-19' vs. 'Non-COVID-19' at 'Female' vs. 'Male'			1.50 (1.11, 2.03)	
**COVID-19 Status (ref = Non-COVID-19) by Income (ref = ≥$100,000)**				
'Probable/Confirmed COVID-19' vs. 'Non-COVID-19' at '<$50,000' vs. '≥$100,000'				
'Probable/Confirmed COVID-19' vs. 'Non-COVID-19' at '≥$50,000 - <$100,000' vs. '≥$100,000'				
'Suspected COVID-19' vs. 'Non-COVID-19' at '<$50,000' vs. '≥$100,000'				
'Suspected COVID-19' vs. 'Non-COVID-19' at '≥$50,000 - <$100,000' vs. '≥$100,000'				
**COVID-19 Status (Non-COVID-19) by Number of Chronic Conditions (ref = <3)**				
'Probable/Confirmed COVID-19' vs. 'Non-COVID-19' at 'Female' vs. 'Male'				3.28 (0.91, 11.83)
'Suspected COVID-19' vs. 'Non-COVID-19' at 'Female' vs. 'Male'				5.34 (4.06, 7.02)

© 2022 Beauchamp MK et al. *JAMA Network Open.*
