# Supplementary material for: Assessment of Functional Mobility After COVID-19 in Adults Aged 50 Years or Older in the Canadian Longitudinal Study on Aging
Source: JAMA Netw Open. 2022 Jan 12;5(1):e2146168. doi: 10.1001/jamanetworkopen.2021.46168 (PMC8756318; doi:10.1001/jamanetworkopen.2021.46168)
Supplement: Supplement 2. — The Canadian Longitudinal Study on Aging Team Members [file jamanetwopen-e2146168-s002.pdf]

Supplemental Online Content: Nonauthor Collaborators

*Indicates required information. Only first name, last name, and suffix will appear in PubMed.

***Group Name(s): Canadian Longitudinal Study on Aging (CLSA) Team**							
***First Name and Middle Initial(s)**	***Last Name**	***Suffix (eg, Jr, III)**	Academic Degrees	Institution	Location (city, state/province, country)	Role or Contribution, eg, chair, principal investigator	Group (if more than 1 Group listed in the byline) and/or Subgroup (eg, Steering Committee)
Andrew	Costa		PhD	McMaster University	Hamilton, Ontario, Canada	Local Site Principal Investigator	
Laura	Anderson		PhD	McMaster University	Hamilton, Ontario, Canada	Local Site Principal Investigator	
Cynthia	Balion		PhD	McMaster University	Hamilton, Ontario, Canada	Local Site Principal Investigator	
Asada	Yukiko		PhD	Dalhousie University	Halifax, Nova Scotia, Canada	Local Site Principal Investigator	
Benoît	Cossette		PhD	University of Sherbrooke	Sherbrooke, Quebec, Canada	Local Site Principal Investigator	
Melanie	Levasseur		PhD	University of Sherbrooke	Sherbrooke, Quebec, Canada	Local Site Principal Investigator	
Scott	Hofer		PhD	University of Victoria	Victoria, British Columbia, Canada	Local Site Principal Investigator	
Theone	Paterson		PhD	University of Victoria	Victoria, British Columbia, Canada	Local Site Principal Investigator	
David	Hogan		MD	University of Calgary	Calgary, Alberta, Canada	Local Site Principal Investigator	
Teresa	Liu-Ambrose		PhD	University of British Columbia	Vancouver, British Columbia, Canada	Local Site Principal Investigator	
Verena	Menec		PhD	University of Manitoba	Winnipeg, Manitoba, Canada	Local Site Principal Investigator	
Philip	St. John		MD	University of Manitoba	Winnipeg, Manitoba, Canada	Local Site Principal Investigator	
Gerald	Mugford		PhD	Memorial University of Newfoundland	St. John's, Newfoundland and Labrador, Canada	Local Site Principal Investigator	

Supplemental Online Content: Nonauthor Collaborators

*Indicates required information. Only first name, last name, and suffix will appear in PubMed.

***First Name and Middle Initial(s)**	***Last Name**	***Suffix (eg, Jr, III)**	Academic Degrees	Institution	Location (city, state/province, country)	Role or Contribution, eg, chair, principal investigator	Group (if more than 1 Group listed in the byline) and/or Subgroup (eg, Steering Committee)
Zhiwei	Gao		PhD	Memorial University of Newfoundland	St. John's, Newfoundland and Labrador, Canada	Local Site Principal Investigator	
Vanessa	Taler		PhD	University of Ottawa	Ottawa, Ontario, Canada	Local Site Principal Investigator	
Patrick	Davidson		PhD	University of Ottawa	Ottawa, Ontario, Canada	Local Site Principal Investigator	
Andrew	Wister		PhD	Simon Fraser University	Vancouver, British Columbia, Canada	Local Site Principal Investigator	
Theodore	Cosco		PhD	Simon Fraser University	Vancouver, British Columbia, Canada	Local Site Principal Investigator	
